# Microbiome Responses to an Uncontrolled Short-Term Diet Intervention in the Frame of the Citizen Science Project

**DOI:** 10.3390/nu10050576

**Published:** 2018-05-08

**Authors:** Natalia S. Klimenko, Alexander V. Tyakht, Anna S. Popenko, Anatoly S. Vasiliev, Ilya A. Altukhov, Dmitry S. Ischenko, Tatiana I. Shashkova, Daria A. Efimova, Dmitri A. Nikogosov, Dmitrii A. Osipenko, Sergey V. Musienko, Kseniya S. Selezneva, Ancha Baranova, Alexander M. Kurilshikov, Stepan M. Toshchakov, Aleksei A. Korzhenkov, Nazar I. Samarov, Margarita A. Shevchenko, Alina V. Tepliuk, Dmitry G. Alexeev

**Affiliations:** 1 Knomics LLC, Skolkovo Innovation Center, Bolshoy Bulvar Str., Building 42, Premise 1, Rooms 1293-1296, Moscow 143026, Russia; a.tyakht@gmail.com (A.V.Ty.); popenko@atlasbiomed.com (A.S.P.); anatoly.developer@gmail.com (A.S.V.); ilya.altukhov@gmail.com (I.A.A.); ischenko.dmitry@gmail.com (D.S.I.); shashkova@phystech.edu (T.I.S.); dar9468qwerty@gmail.com (D.A.E.); dmitry.g.alexeev@gmail.com (D.G.A.); 2 Computer Technology Department, ITMO University, Kronverkskiy pr., 49, St. Petersburg 197101, Russia; 3 Department of Biological and Medical Physics, Moscow Institute of Physics and Technology, Institutskiy per. 9, Dolgoprudny, Moscow Region 141700, Russia; aancha@gmail.com; 4 Department of Natural Science, Novosibirsk State University, Pirogova Str., 1, Novosibirsk 630073, Russia; 5 Atlas Biomed Group, 92 Albert Embankment, Lambeth, London SE1 7TT, UK; nikogosov@atlas.ru (D.A.N.); osipenko@atlas.ru (D.A.O.); musienko@atlasbiomed.com (S.V.M.); 6 Atlas Medical Center, Kutuzovsky prospekt 34 bld. 14, Moscow 121170, Russia; selezneva@atlas.ru; 7 Research Centre of Medical Genetics, Moskvorechye Str., 1, Moscow 115478, Russia; 8 School of Systems Biology, George Mason University, Fairfax, VA 22030, USA; 9 Department of Genetics, University Medical Center Groningen, University of Groningen, 9712 CP, Groningen, The Netherlands; alexa.kur@gmail.com; 10 School of Life Sciences, Immanuel Kant Baltic Federal University, Universitetskaya Str. 2, Room 106, Kaliningrad 236040, Russia; stepan.toshchakov@gmail.com (S.M.T.); oscypek@yandex.ru (A.A.K.); nazar.sni@gmail.com (N.I.S.); lionsorciere@gmail.com (M.A.S.); aeternusmare1414@gmail.com (A.V.Te.); 11 Winogradsky Institute of Microbiology, Research Centre of Biotechnology RAS, Leninsky prospect 33-2, Moscow 119071, Russia

**Keywords:** gut microbiota, personalized diet, microbiome stability, intervention, 16S rRNA metagenomics, citizen science, responders

## Abstract

Personalized nutrition is of increasing interest to individuals actively monitoring their health. The relations between the duration of diet intervention and the effects on gut microbiota have yet to be elucidated. Here we examined the associations of short-term dietary changes, long-term dietary habits and lifestyle with gut microbiota. Stool samples from 248 citizen-science volunteers were collected before and after a self-reported 2-week personalized diet intervention, then analyzed using 16S rRNA sequencing. Considerable correlations between long-term dietary habits and gut community structure were detected. A higher intake of vegetables and fruits was associated with increased levels of butyrate-producing *Clostridiales* and higher community richness. A paired comparison of the metagenomes before and after the 2-week intervention showed that even a brief, uncontrolled intervention produced profound changes in community structure: resulting in decreased levels of *Bacteroidaceae*, *Porphyromonadaceae* and *Rikenellaceae* families and decreased alpha-diversity coupled with an increase of *Methanobrevibacter*, *Bifidobacterium*, *Clostridium* and butyrate-producing *Lachnospiraceae*- as well as the prevalence of a permatype (a bootstrapping-based variation of enterotype) associated with a higher diversity of diet. The response of microbiota to the intervention was dependent on the initial microbiota state. These findings pave the way for the development of an individualized diet.

## 1. Introduction

The importance of gut microbiota to human health is hard to overestimate. Comparative surveys have revealed associations between some non-communicative diseases and the underrepresentation of certain commensal microbial taxa as well as with the increased prevalence of potential pathobionts [1,2,3,4,5,6]. At the same time, due to the immense variability in microbial composition at the species level, the jury is still out on what may constitute the elusive “golden standard” of a healthy gut [7]. Nevertheless, microbiota modulations rapidly develop as an avenue of personalized medicine. The international randomized study Food4Me recently concluded that individualized nutritional recommendations lead to better health outcomes than a “one-size-fits-all” dietary approach [8]. Another large-scale study showed that microbiota-tailored diets strongly influence a postprandial glycaemic response, thus allowing for the personalized control of metabolic status [9]. However, the durability of the microbiota-driven response to drastic dietary changes is far from being confirmed [10].

A controlled setting allows the researchers to ensure compliance with dietary requirements [11,12,13]. However, there is always a trade-off between a proper experimental control and the ecological validity of the findings. Both novelty of environment and an increase in motivation to comply (known as the Hawthorne effect) are detectable in investigated subjects, and they undermine a real-life generalization of the findings inferred from laboratory-based research [14]. In order to investigate whether gut microbiota composition changes after a short-term dietary intervention under a free-living setting, we used metagenomic sequencing. This technique previously allowed researchers to outline the landscape of a microbiome composition in a series of large-scale international projects [15,16,17] that are now becoming increasingly available to the public in formats ranging from participation in “citizen science” initiatives (e.g., American Gut [18] or µBiome [19]) to the high-frequency sampling of personal microbiota [20,21]. Using an Internet-based crowdfunding platform, 248 subjects were recruited from the urban population (Moscow, Russia). For each individual, stool samples were collected before and after a 2-week personalized diet intervention and analyzed using 16S rRNA sequencing. At the baseline, microbiota composition characteristics were significantly linked to food frequency questionnaire items, body mass index, gender and age. A paired comparison of the metagenomes before and after the dieting showed that even a brief, uncontrolled intervention may produce considerable changes in the composition of microbiota.

## 2. Materials and Methods

### 2.1. Study Design and Sample Collection

The research was approved by the local ethics committee of the Atlas Medical Center, LLC. The project was conducted in accordance with the principles expressed in the Declaration of Helsinki. Volunteers were recruited using an Internet-based crowdfunding platform called Boomstarter (https://boomstarter.ru; a translated information video is available at https://youtu.be/PI7OzBz7ALo) and signed informed consent forms before entering the study. The exclusion criteria are listed in Supplementary Methods. Each volunteer filled in a questionnaire about long-term dietary habits (food frequency questionnaire), lifestyle, medical and anthropometric factors (Table S1, Supplementary Methods). Food product consumption frequency was assessed as the number of intakes per month. The volunteers were instructed on how to perform sample collection at home. According to the instructions, fecal samples should be collected and immediately placed into a freezer individually by each of the volunteers. The frozen samples were transported to the laboratory on ice, with the transportation time not greater than 2 h. After collecting an initial (baseline) sample, the volunteers were provided with dietary recommendations essentially targeting a more balanced diet, in part by increasing fiber content. Dietary recommendations are listed in Table S2. For each participant, they consisted of the general part (identical for all participants) and individual part (based on the participant’s answers to questionnaire). The general part included eating more vegetables and fruit, reducing amount of sugar, salt, saturated fats and “empty calories” as well as distributing food intake more evenly through the day. The individual part was compiled based on the results of the questionnaire according to the algorithm described in Table S3. The algorithm aims to increase the consumption of specific healthy food products underrepresented in the volunteer’s long-term diet and to decrease the consumption of overrepresented “junk foods”. After two weeks of following the recommendations in an uncontrolled environment, each volunteer collected the second fecal sample.

The control group—7 subjects who did not change their diet—followed the same sample collection procedure (like in the test group, the control samples were collected 2 weeks apart).

### 2.2. Calculation of Sample Size

The sample size analysis was based on the hypothesis that the described diet intervention significantly affects microbiota community structure and the choice of pairwise PERMANOVA as a method for testing this hypothesis. In order to identify the minimal number of subjects required to achieve the statistical power of 80% to detect the pre-specified effect size, we applied a framework for PERMANOVA power estimation from the micropower R package [22]. For this analysis, we estimated the population distribution of pairwise dissimilarity (mean = 0.52, s.d. = 0.06) using the published data on the gut microbiota of healthy Russian subjects [1] (*n* = 61 samples) and the expected effect size (ω^2^ = 0.006)—using the data from a study of a short-term high-fiber dietary intervention [13] (*n* = 10 samples). The resulting required sample size was 70 subjects (see Figure S1). The total number of the volunteers who took part in the crowdfunding was about three times higher, resulting in power of >99%.

### 2.3. Fecal Sample Preparation and Metagenomic Analysis

The extraction and sequencing of DNA is described in the Supplementary Methods. Amplicon sequencing of the V4 variable region of the microbial 16S rRNA gene was performed using a MiSeq sequencer (Illumina, San Diego, CA, USA). The reads were analyzed in QIIME v.1.7.0 [23] using the HITdb v. 1.0 database [24] for taxonomic assignment (see Supplementary Methods). Prediction of metabolic potential profiles was performed using Greengenes v. 13.5 database [25] and PICRUSt [26].

### 2.4. Data Availability

The raw reads were deposited in the European Nucleotide Archive (project accession ERP018192).

### 2.5. Statistical Analysis

All statistical analysis was performed in R statistical programming language, version 3.3.0 [27]. Data preprocessing steps are described in the Supplementary Methods section.

#### 2.5.1. Identification of the Links between Microbiota Composition and Metadata

Analysis was performed on 207 subjects for whom the questionnaire data was available (see Supplementary Methods). The associations between each of the factors included in the metadata and general microbiota composition were estimated using a permutational multivariate analysis of the variance using distance matrices implemented in the adonis function from the vegan package [28] with generalized UniFrac distance [29]. During analysis with the adonis function, the number of permutations was 2000. Categorical factors were tested for homoscedasticity using the PERMDISP2 method implemented in the betadisper function from the vegan package. The associations between each factor and alpha-diversity were identified using the Spearman correlation. Multiple comparison correction for the *p*-values was performed using the Benjamini–Hochberg method (here and elsewhere).

The links between individual microbial taxa and factors were identified using a general linear model approach implemented in the MaAsLin [30] R package. Analysis was performed separately for each of the factors; the significant factors according to the adonis results were included in each model for correction. The boosting step was skipped. Low-abundant taxa (present at less than 0.2% of total microbial abundance in more than 15 samples) were filtered out. The significance criterion for MaAsLin was: adjusted *p* < 0.1.

#### 2.5.2. Differential Analysis of Microbial Taxa and Functions

Analysis was performed on 430 samples from 215 subjects. The taxa for which the relative abundance significantly changed after the intervention were identified using paired analysis in the metagenomeSeq package [31]; validation was performed by applying a compositionality-aware ALDEx2 [32] algorithm (with centered-log ratio transformation, Wilcoxon signed-rank test and Benjamini–Hochberg *p*-value adjustment). The effect size of change for each of the identified taxa was calculated using the LEfSe method [33]. The changes were considered significant if the logarithm (base 10) of the effect size was above 2.0 and the adjusted *p*-value was below 0.05. Differential analysis of the predicted metabolic potential of microbiota was performed using the piano R package [34] in a paired fashion with the following parameters: gene set analysis method: “reporter features”, significance threshold: adjusted *p* < 0.05.

#### 2.5.3. Identification of Responders among the Volunteers

In order to investigate the individual response to short-term diet intervention, the subjects were clustered based on the dissimilarity between their paired samples calculated using a generalized UniFrac metric [29]. The clustering procedure was performed with partitioning around the medoids with estimation of number of clusters algorithm implemented in the fpc R package [35] (pamk function) with a range of cluster numbers from 2 to 10. The optimal number of clusters (2) was selected as the one that maximizes the average silhouette width of the clusters. Subjects from a cluster with lower value of mean dissimilarity were named “non-responders” and subjects from a cluster with greater value of mean dissimilarity were named “responders”. The taxa differentially abundant in the microbiota of “responders” and “non-responders” were identified using metagenomeSeq. Associations between the group and questionnaire results as well as personalized recommendations were identified using the chi-square test (for categorical and logical factors) and the Mann–Whitney test (for quantitative factors).

The random forest classifier was used to predict if a subject belonged to the “responders“ or “non-responders“ group based on the baseline microbiota composition. R package caret [36] was used for training and testing the classifier and the ROCR [37] package was used for obtaining its performance estimations. Only the significantly different features between responders and non-responders at the baseline were included in the classifier as predictors. The classifier was created and tested separately at each taxonomic level. Cross-validation random sampling was performed 10 times for 70% of samples for the train dataset and 30% for the test dataset to benchmark the classification quality. For each iteration, a ROC curve was plotted and AUC was calculated. Mean values of all the iterations were calculated.

#### 2.5.4. Cluster Analysis of Samples and Microbial Taxa

Microbial cooperatives were identified using the SPIEC-EASI package, a compositionally robust technique to analyze bacterial networks [38]. The cooperatives were obtained using the complete set of metagenomes (in a control analysis, when only the baseline samples were used, similar cooperatives were produced). Genera having less than 10 reads per sample on average were excluded. In the SPIEC-EASI algorithm, neighbors were selected using the Meinshausen and Bühlmann method, while the model selection was performed using the StARS algorithm (huge R package [39]) (number of subsamples = 50, number of lambda iterations = 20, minimum lambda ratio = 0.1). Cooperative was defined as a connected component of the co-occurrence graph with more than two vertices.

Clustering of the metagenomes was performed using the original enterotyping algorithm [40] as well as its novel bootstrapping-based modification, called permatyping (see Supplementary Methods). For enterotyping and permatyping, 416 samples from 222 subjects were used. These samples excluded subjects who reported to have lactose or gluten intolerance. Original enterotyping [40] was performed at the genus level using the Jensen–Shannon metric for distance matrix calculation, the PAM clustering algorithm, and the optimal number of clusters was selected by maximizing the Calinski–Harabasz index. Associations between the obtained clusters (permatypes) and factors were identified: for quantitative factors, using the Mann–Whitney test; for categorical and logical factors, using the chi-square test.

## 3. Results

### 3.1. Variation of Gut Microbiota in the Urban Population

Totally 260 subjects initially enrolled to the study, both samples before and after the diet intervention were provided by 248 subjects, thus demonstrating a relatively high compliance. After preprocessing 16S rRNA metagenomic reads and removing the subjects with low coverage in one or both metagenomes, a total of 215 pairs of metagenomes remained. More than 93% of resultant reads were successfully classified, signifying the validity of the selected technique of composition profiling.

In the examined cohort, microbiota was found to be quite diverse, with individual samples generally falling into previously reported community structures of the Russian population obtained using “shotgun” metagenomics [17] (Figure S2). A total of 42 families, 122 genera, and 692 species were detected in at least one sample (the complete table of relative abundance is listed in Table S4). Overall, the most represented phyla were *Firmicutes* (83.4 ± 9.7%), *Bacteroidetes* (6.8 ± 8.7%), and *Actinobacteria* (3.4 ± 4.5%).

### 3.2. Long-Term-Dietary Habits, Antibiotic Intake and Anthropometric Indices Are among the Major Factors Associated with Microbiota Composition

We analyzed the associations between the gut community structure of the volunteers and various factors obtained from the questionnaires, including self-reported long-term dietary habits, lifestyle indicators, anthropometric indices, and medications (see Table S1). Questionnaire results are available for 207 subjects (see Supplementary Notes). Distribution of the factors from the questionnaire are listed in Table S5. The cohort included 97 women (24–61 years old) and 110 men (18–64 years old). The BMI was 24.2 ± 4.9 (median ± s.d.): 15 subjects had low BMI (<18.5), 17—first-degree obesity (30–34.9), 6—second-degree obesity (35-39.9) and one subject—third-degree obesity (≥40).

The largest contributions to the overall microbiota composition (see Supplementary Methods) were detected for intake of antibiotics within the last three months (percentage of the total variance explained by the factor 1.41%, pseudo-F = 2.93), dairy consumption (1.12%, pseudo-F = 2.47), and gender (1.11%, pseudo-F = 2.58) (*n* = 207 subjects, FDR adjusted *p* < 0.05); see Figure S3.

Analysis of the links between assessed factors and the richness of microbiota (see Supplementary Methods) showed that, in the subjects who took antibiotics within the last three months, alpha-diversity was significantly decreased. On the other hand, alpha-diversity was positively linked to the amount of vegetables in long-term dietary habits (Spearman correlation coefficients of −0.2 and 0.2, respectively, *n* = 207 subjects, *p* < 0.03, FDR adjusted *p* < 0.05). We also observed a suggestive negative correlation between alpha-diversity and BMI (*n* = 207 subjects, alpha-diversity measured via chao1 index, *p* = 0.02, FDR adjusted *p* = 0.09) (Figure S4).

**Figure 1:**
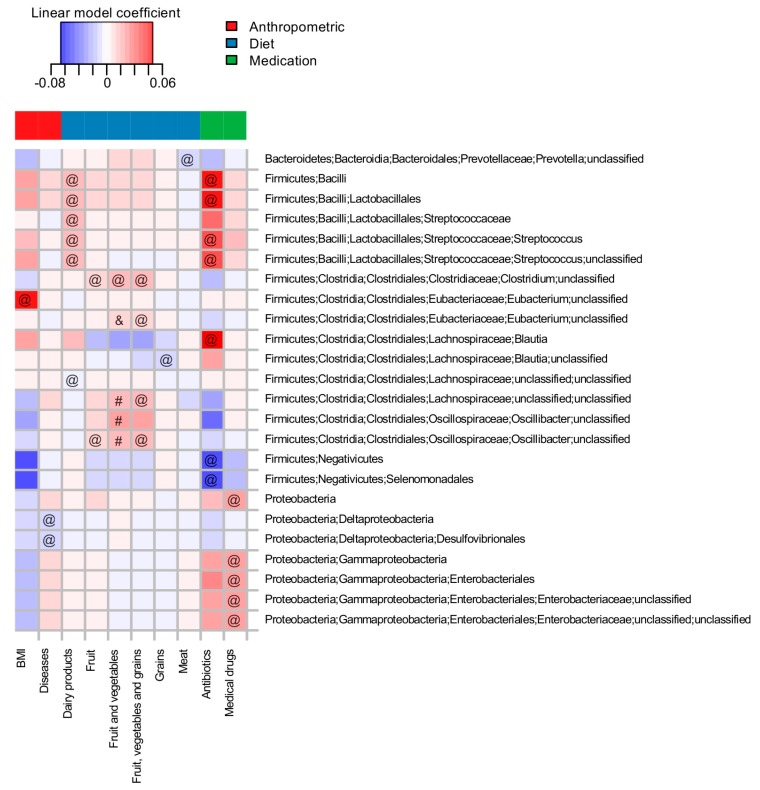
Associations of the microbial taxa with long-term dietary habits and other factors from the questionnaire (*n* = 207 subjects). Analysis was performed for the baseline samples at taxonomic levels from species to phyla. Rows are sorted in alphabetic order. Cell color denotes the value of the linear model coefficient from the MaAsLin analysis. All significant associations (FDR adjusted *p* < 0.1) are marked with one of the symbols (&, #, @): “&”—associations previously reported by Zhernakova et al., 2016 [41], “#”—reported by Wu et al., 2011 [13], “@”—novel associations.

An adjustment for the effects of intake of antibiotics, the frequency of dairy consumption and gender revealed links between the relative abundance of individual taxa and a number of the factors. Significant associations are listed in Figure 1 and Table S6. It is of note that several associations known from larger studies were replicated, while several new associations were uncovered (Figure 1).

The factors significantly associated with individual taxa were generally similar to those factors uncovered by variance analysis to include antibiotic intake, medicinal drug intake, chronic diseases, gender, body mass index, frequency of consumption of dairy products, fruit, total vegetables and fruit, and grains and meat (*n* = 207 subjects, FDR-adjusted *p* < 0.1).

### 3.3. Short-Term Dietary Changes Significantly Shift Microbiota Composition

Volunteers have received dietary recommendations according to their long-term dietary habits, assessed using a questionnaire (see Supplementary Methods section and Tables S2 and S3). A post-hoc analysis of individual sets of recommendations revealed a key piece of advice assigned to a majority of the participants. This advice was to increase fiber consumption and avoid habitual consumption of the “Western diet”. In a sense, this advice was narrowing a variation in the volunteers’ diets (see Figure S5).

The changes in the microbiota composition at the end of the 2-week period when the participants followed the recommendations were significant (pairwise PERMANOVA, *n* = 430 paired samples, *p* = 0.0005, 4.17% of the total variation explained, pseudo-F = 18.61; see Figure S6; paired sample identifiers are listed in Table S7). At the individual level, the shifts were quite dramatic (Bray–Curtis index 0.45 ± 0.11 between paired metagenomes), being higher than technical (0.22 ± 0.02 between technical replicates at the level of DNA extraction) and lower than group-wise variation (0.68 ± 0.08 between random metagenomes). Notably, for the members of the voluntary control group that did not change their diet, over the course of two weeks these changes were substantially less pronounced (Bray–Curtis index 0.26 ± 0.08, *n* = 7 subjects—see Supplementary Methods and Figure S7).

Interestingly, a slight but significant decrease of alpha-diversity in dieting volunteers was observed—the average fold-change was 1.04 (Shannon index changed from 5.74 ± 0.52 to 5.53 ± 0.54, Welch’s test *p* = 8.2 × 10^−5^, *n* = 430 samples). This effect persisted with the other diversity metrics as well as the rarefaction depth (see Figure S8).

**Figure 2:**
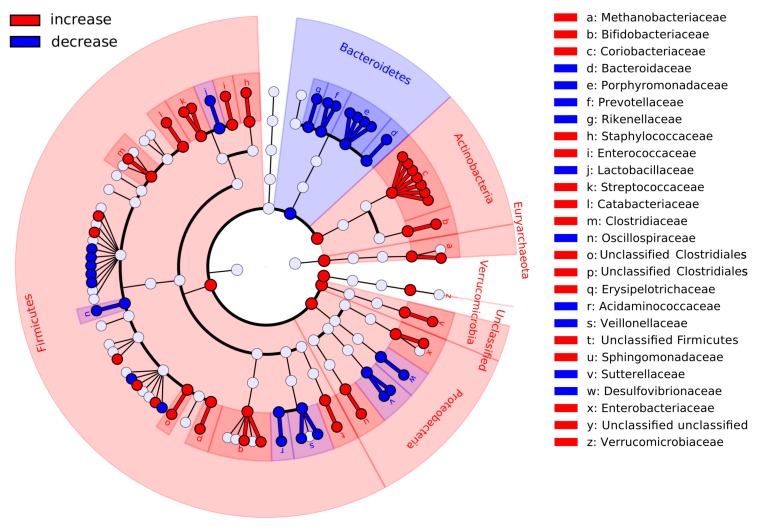
Major changes in the gut community structure of the volunteers after following the dietary recommendations. Red branches of the cladogram denote the taxa that were increased in abundance, while the blue ones—decreased. Significance criterion: *p* < 0.05 in metagenomeSeq model and log10 of the effect size >2 in LEfSe method (*n* = 430 paired samples).

Diet-associated changes in relative abundance for each of the microbial taxa were analyzed from the levels of phyla down to species (see Supplementary Methods). The most global changes—at the levels of phyla and families—are shown in Figure 2, with the complete results presented in Table S8. There was a decrease in the abundance of many genera from the *Bacteroidetes* phylum; this was accompanied by an increase for microbes from *Actinobacteria*, *Firmicutes* as well as from *Euryarchaeota*. The results were similar when a compositionality-aware approach [32] was applied (see Supplementary Methods, Figures S9 and S10, Table S9). The *Bacteroidetes*:*Firmicutes* ratio significantly decreased (from 0.13 ± 0.2 to 0.03 ± 0.09, Wilcoxon paired test *p* < 0.0001, *n* = 430 paired samples, see Figure S11). Analysis of metabolic potential also showed depletion of many functions inherent to members of the *Bacteroidetes* (see Tables S10 and S11), including the biotin and riboflavin biosynthesis pathways modules (M00125, M00572).

### 3.4. The Subjects Vary in How Gut Microbiota Responds to Dietary Intervention

**Figure 3:**
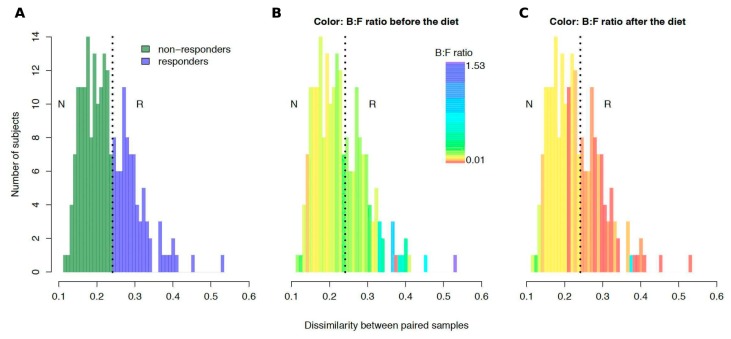
Interindividual variation of gut microbiota response to the diet intervention (*n* = 215 subjects). Distribution of taxonomic dissimilarity between the metagenomes before and after the intervention for each subject (generalized UniFrac metric) is colored in different ways. In panel (**A**), the color denotes responders (blue) and non-responders (green). In panels (**B**) and (**C**) the color denotes the average *Bacteroidetes*:*Firmicutes* ratio for the samples collected before and after the diet, respectively). Abbreviations: B:F ratio—*Bacteroidetes*:*Firmicutes* ratio, N—non-responders, R—responders.

An analysis of pairwise dissimilarity of community structures before and after the intervention revealed the bimodal nature of the changes observed. Clustering of the subjects by the magnitude of changes (see Supplementary Methods) yielded 2 clusters: “non-responders” with relatively stable microbiota (*N* = 130; distance between paired points 0.19 ± 0.03) and “responders” with less stable microbial communities (*N* = 85; distance 0.30 ± 0.05) (Figure 3A, Table S12). No significant associations of clusters were detected, neither with any specific recommendation nor with metadata extracted from the questionnaire (see Supplementary Methods, FDR-adjusted *p* > 0.1, *n* = 175 subjects).

However, at the baseline, the gut microbiota of the “responders” was distinct from that of the “non-responders” (see Table S13). “Responders” had lower fractions of *Actinobacteria* (*Coriobacteriales* order), *Firmicutes* (*Bacillales*, *Erysipelotrichales* and *Clostridiales* order), *Proteobacteria* (*Enterobacteriales* order), and *Verrucomicrobia* (*Verrucomicrobiales* order) phyla, while the *Bacteroidales* and *Sphingomonadales* orders were represented at higher levels. In the “responders” cohort, baseline *Bacteroidetes*:*Firmicutes* ratios were significantly higher than in the “non-responders” (Mann-Whitney test, *p* = 0.0001, *n* = 215) (Figure 3B). As expected, the metabolic modules and pathways enriched in microbiota of “responders” included the modules specific to Gram-negative microbes, lipopolysaccharide biosynthesis (md:M00060) and NADH:quinone oxidoreductase (md:M00144), while in “non-responders”, *Firmicutes* and *Actinobacteria*-driven enrichment in ABC-transporters (ko02010) was seen [42]. Additionally in “responders”, a “Other glycan degradation pathway” (ko00511) related to the degradation of the carbohydrate components of the gut mucus was enriched.

Interestingly, when the stool samples of the “responders” were analyzed post-diet, the trends for a majority of the mentioned orders (*Coriobacteriales*, *Bacillales*, *Enterobacteriales*, *Bacteroidales*) were reversed (see Table S14, Figures S12 and S13). Furthermore, “responders” also dropped the *Bacteroidetes*:*Firmicutes* ratio after the diet to significantly lower levels than the “non-responders” (*p* = 0.0003, *n* = 215 subjects) (Figure 3C).

To investigate whether the baseline microbiota state of a subject can be used to predict the “responder”/“non-responder” status, a random forest classifier was constructed based on microbial markers that significantly differ in abundance between the groups. The classifiers were also constructed separately for each taxonomic level (Table S15). The performance of classifiers was assessed using repeated random sub-sampling cross-validation (training set *N* = 150, test set *N* = 65) (see Supplementary Methods). The best average AUC value (0.78) was obtained at the species level (Figure S14, Table S15).

### 3.5. Co-Occurring Groups of Microbes Associated with Long- and Short-Term Dietary Factors

For a more comprehensive exploration of the associations between gut microbiota and various characteristics, the dimensionality of the analysis was reduced by clustering, performed at first for microbial genera, then for the samples.

**Figure 4:**
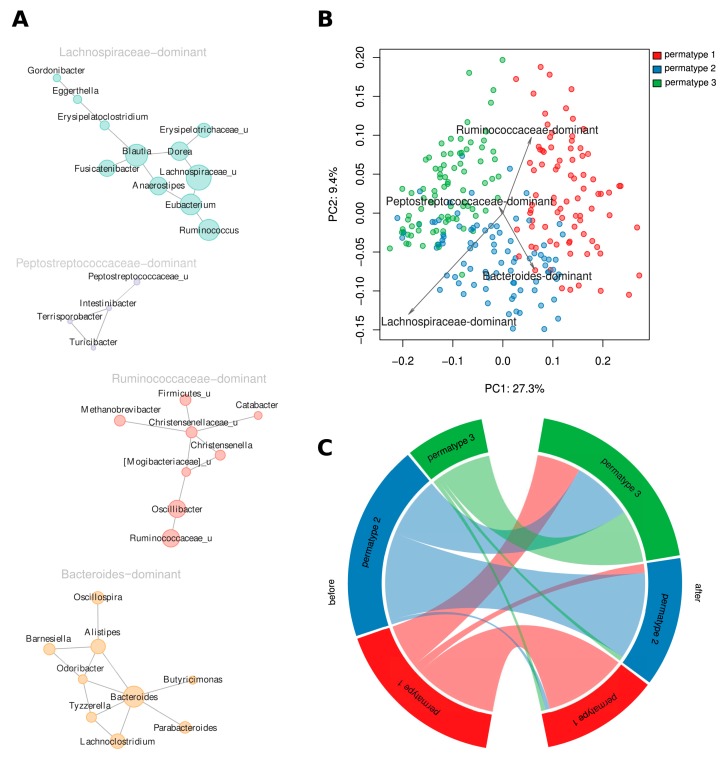
Cluster analysis for microbial genera and samples. (**A**) Cooperatives of microbial genera. Size of the vertices is proportional to the average relative abundance of the genera in all metagenomes. Postfix “_u” denotes all unclassified genera from the respective family. (**B**) Links between cooperatives and permatypes (principal coordinates analysis [PCoA] using generalized UniFrac metric). (**C**) Changes in distribution of the participants across permatypes after following the dietary recommendations.

At the level of genera, four large “cooperatives”, the groups of co-occurring genera representing potentially symbiotic subcommunities (see Supplementary Methods), were identified (Figure 4A, Table S16, Figure S15). These “cooperatives” were denoted as *Lachnospiraceae*-, *Peptostreptococcaceae*-, *Ruminococcaceae*-, and *Bacteroides*-dominant according to their respective major driver taxa.

Identified cooperatives suggest possible functional interactions between gut microbes. *Lachnospiraceae*-dominant cooperative is likely to be formed by the cross-feeding of microbial species specialized in breaking down complex carbohydrates of plant origin, including cellulose and resistant starch (*Ruminococcus*, *Eubacterium*) and the bacteria producing the butyrate from secondary glycans (*Anaerostipes*, *Fusicatenibacter*) [43,44]. Acetogenic bacteria (like *Blautia*) also benefit from cohabiting with primary plant degraders, as they consume hydrogen, a product of glycan fermentation [45].

Similar mutualistic patterns may be proposed for the *Ruminococcaceae*-dominant cooperative, where the hydrogen generated in the process of carbohydrate fermentation (by *Ruminococcus*) is consumed by methanogenic *Archaea* (*Methanobrevibacter*) [43,46]. *Methanobrevibacter* and *Christensenella* were previously observed to be co-occurring [47], both are positively associated with low BMI [47,48,49] and negatively, with an unfavorable lipid profile [50].

In a *Bacteroides*-dominant cooperative, a number of member genera is associated with diets rich in animal protein [51] and includes *Alistipes*, *Bacteroides*, *Barnesiella,* and *Parabacteroides* species, some of which are known to be bile-tolerant [43]. An inclusion of *Oscillospira* in this cooperative may be due to cross-feeding effects through the use of the fermentation by-products of *Bacteroides* [52].

The *Peptostreptococcaceae*-dominant cooperative included several genera from this family: *Intestinibacter*, *Terrisporobacter*, and unclassified ones, as well as *Turicibacter*. The members of the cooperative are generally relatively rare in gut community. The high heritability of *Turicibacter* as well as its co-occurrence with *Peptostreptococcaceae* were previously reported [53].

Analysis of associations between the questionnaire items and relative abundances for each of the four cooperatives showed that the subjects who had recently taken antibiotics had an increased presence of the *Lachnospiraceae*-dominant cooperative but a decreased presence of the *Ruminococcaceae*-dominant cooperative. Female subjects tended to have increased levels of the *Bacteroides*-dominant cooperative as compared to males (MaAsLin method, *p* < 0.1, *n* = 207 subjects). The short-term diet intervention was associated with the decrease of *Bacteroides*-dominant and the increase of *Lachnospiraceae*-dominant cooperatives (Wilcoxon signed-rank test, adjusted *p* = 2.50 × 10^−20^ and 3.9 × 10^−5^, respectively, *n* = 430 paired samples).

### 3.6. Both Short-Term Dietary Intervention and Long-Term Dietary Habits Are Reflected in the Clustering of Community Structures

For the cluster analysis at sample level, we applied the enterotyping methodology [40] and yielded a total of three enterotypes. It should be noted that a number of studies suggested the entorotyping results are to be approached with caution [54]. For the purpose of this study, we developed and applied a bootstrapping-based variation of the enterotyping technique, which we called permatyping (see Supplementary Methods). Accordingly, the obtained clusters of metagenomes were designated as permatypes (Figure S16, Table S17). Briefly, permatyping shrinks the original enterotypes to include only the samples that certainly belong to the clusters, while unstable samples are discarded as unclassified (see Supplementary Methods).

In this study, permatyping produced three clusters including 83, 86, and 81 metagenomes, respectively, while 166 unstable samples were excluded from further analysis. The distinctive microbial genera (drivers) of Permatype 1 included *Oscillibacter* and *Prevotella*; Permatype 2 included unclassified genera from *Lachnospiraceae*, *Roseburia*, and *Bacteroides*; and Permatype 3 included *Dorea*, *Blautia* and *Staphylococcus* (Figure S17). These sets of drivers somewhat resembled the ones described for the originally discovered enterotypes [40] by also including *Bacteroides* and *Prevotella* for two different permatypes. Obviously, their lower ranking was linked to the fact that our sample included few of the metagenomes enriched in *Bacteroides* and *Prevotella* identified in some other studies [55]. The relative abundance of the microbial cooperatives of all three permatypes were compared (Figure 4B, Figure S18). Permatype 1 samples had increased levels of the *Ruminococcaceae*-dominant cooperative (Mann–Whitney test, adj. *p* = 1.2 × 10^−4^, *n* = 250 samples). Permatype 2 was enriched in the *Lachnospiraceae*- and *Bacteroides*-dominant cooperatives (*p* = 1.5 × 10^−22^, *n* = 250 samples). Permatype 3 was enriched in the *Lachnospiraceae*-dominant cooperative (*p* = 6.8 × 10^−26^, *n* = 250 samples). A comparison of microbial diversity between permatypes showed that the samples of Permatype 1 had significantly higher diversity than each of the two others (Shannon index 6.08 ± 0.51 vs. 5.32 ± 0.50 and 5.51 ± 0.47, respectively, one-sided Welch’s test *p* < 0.05, *n* = 250 samples).

In the analysis of variations in long-term dietary habits between the enterotypes and the permatypes of microbiota, no significant differences were detected for the original enterotypes. However, for permatypes, some associations were uncovered. Baseline profiles for the three permatypes included 47, 49, and 18 metagenomes. The dietary habits and demographics of Permatype 1 individuals did not manifest significant differences from that of the entire cohort, while Permatype 2 individuals were, on average, younger (29 ± 7 vs. 33 ± 9 years) (adj. *p* < 0.1, *n* = 114 subjects) and consumed less vegetables and fruits than the other participants. The members of Permatype 2 also consumed more meat and beer and less fish and seafood (see Table S18, Figure S19). Overall, Permatype 2 diet tended to be less diverse: the reported variety of consumed foods was lower. Permatype 3 was less distinct in terms of questionnaire data. Its members’ diet was more diverse (adj. *p* < 0.1, *n* = 114 subjects) and they tended to consume more vegetables (adj. *p* = 0.13, *n* = 114 subjects).

After a short-term dietary intervention, the distribution of subjects among the permatypes changed. The dynamics of transitions between the permatypes (160 paired samples from 80 subjects, Figure 4C) showed that around a third of the subjects initially belonging to Permatypes 1 and 2 moved to Permatype 3 as a result of dieting. The others preserved their permatype. The subjects initially belonging to Permatype 3 (*n* = 12 of 14 subjects) tended to reside in this permatype after the diet, although at the edge of significance, possibly due to low amounts of paired samples of Permatype 3 (Fisher’s test for permatype 3 vs. other samples, resided in initial permatype, *p* = 0.055, *n* = 89 subjects).

## 4. Discussion

A growing body of evidence accumulated by studies of gut microbiota in world populations emphasizes that lifestyle and especially diet strongly impact microbiota composition and, thus, human health. However, it is still unclear how durable are the effects of dietary changes, either long- or short-term. Effects of self-administered short-term dieting efforts on gut microbiota are also far from being well understood. To investigate this problem, we used an Internet-based citizen science-supportive platform to enroll individuals from the Russian urban population into the study aimed at identifying the links between gut metagenome composition and long-term dietary habits, assessed using a food frequency and lifestyle questionnaire and short-term dietary changes achieved during a 2-week intervention.

One of the major observations derived from the analysis of microbiota composition and the recent medical history of the subjects was an influence exerted on gut community by recent intake of antibiotics. No detailed breakdown of antimicrobial drugs was available. The diversity of the antibiotics modes of action might explain the lack of consistency in observations concerning individual microbial taxa. However, on the level of cooperatives, or symbiotic groups of microbes, a significant depletion of the *Ruminococcaceae*-dominant cooperative was detected in the gut of the participants recently exposed to antibiotics. Interestingly, many drivers of this antibiotic-sensitive cooperative, in particular *Methanobrevibacter* and *Christensenella*, were previously linked to leanness. These bacterial generally also manifest high heritability [47]. Another member of this cooperative, *Oscillibacter*, is associated with normal BMI [56]. Moreover, many microbes from this cooperative, including *Ruminococcaceae* and *Oscillibacter*, are known to degrade complex dietary fibers, resulting in the production of butyrate [57], an anti-inflammatory compound that also plays essential roles in the regulation of metabolism, glucose tolerance, and gut motility [58]. These observations support our findings that the abundance of the *Ruminococcaceae*-dominant cooperative correlates with the long-term trends in the consumption of fruit and vegetables.

The idea that antibiotics-driven disruption of the ability of microbiota to support host metabolism contributes to the risk of metabolic diseases, especially in the younger ages, has been discussed before [59,60,61]. It is tempting to speculate that antibiotics might interfere with the host metabolism, through the depletion of beneficial microbial taxa with slow recovery rates and high heritability. In turn, the loss of these specialists would render the host less responsive to the microbiota-mediated effects of high-fiber diet interventions, and, therefore, less likely to return back to the original, “healthier” state.

Out of all long-term diet features, fiber content was the most prominently associated with microbiota composition. The list of microbes correlated with the consumption of fruit, vegetables, and grains included taxa actively involved in the degradation of non-digestible polysaccharides, in particular, species belonging to *Oscillibacter* [62], *Eubacterium* [42], *Blautia* [63] and ones related to *Clostridium clariflavum* [64]. On the other hand, the consumption of meat products was inversely correlated with the abundance of unclassified species from *Prevotella*, a genus linked to diets low in animal protein and high in fiber [65,66]. Subjects who consumed more dairy products had higher levels of *Streptococcus* in their microbiota, possibly because *S. thermophilus*, a major component of starter cultures for fermented milk products, is capable of survival in the human gut [67].

Gender was the only anthropometric factor significantly associated with microbiota composition (Figure S3). *Bacteroides*-dominant cooperative was increased in the gut of female participants. This agrees with the previous observations that harder stools are more common in women, and that harder stools have higher fraction of *Bacteroides* than loose samples [68].

In our study, the dietary change recommendations were quite general, with predominant targeting of the fiber consumption, and an adherence to the recommendations was uncontrolled. Nevertheless, paired comparison of gut metagenomes before and after the 2-week diet intervention detected substantial changes in the structure of the gut community. In voluntary dieters, the magnitude of observed change was about two times higher than that in subjects who did not change their diet, and several times higher than the technical variation introduced at the stages of DNA extraction, sample collection, and library preparation (Figure S7).

While some of the identified short-term changes in the microbial landscape resembled the impacts of long-term high-fiber diet, others were specific and novel. In particular, there was a significant decrease of the *Bacteroides*-dominant cooperative as well as of many of its members, including *Bacteroides* and *Alistipes* and in the related *Bacteroidaceae*, *Porphyromonadaceae* and *Rikenellaceae* families. Many microbes from these taxa are either bile-tolerant or previously positively associated with long- or short-term diets rich in animal protein and saturated fats [11,69,70]. Apparently, due to intervention-related increase of the fiber intake and, possibly, to partial replacement of animal products with the fiber-containing ones, these bacteria yielded to those specializing in a variety of complex polysaccharides. The microbes that increased after the diet included those associated with a healthy gut to include *Clostridiaceae*, particularly, *Clostridium* genus, previously linked to high-fiber diet [71]. *Methanobrevibacter* and *Bifidobacterium* were reported to be inversely correlated with BMI [48] and *Lachnospiraceae*-dominant cooperative was enriched with butyrate producers (*Dorea*, *Ruminococcus* and *Eubacterium*). Interestingly, we also observed a decrease of *Prevotellaceae* (on the genus level—of *Prevotella*) associated with the long-term consumption of high-fiber diet.

Changes at the microbial taxa level were mirrored by transitions between permatypes (Figure 4C). After the diet, many subjects originally belonging to Permatypes 1 and 2 moved to Permatype 3, while a majority of Permatype 3 subjects maintained their permatype. Having a Permatype 3 was associated with higher consumption of vegetables, higher diversity of diet, and high prevalence of butyrate-producing *Firmicutes*. Overall, we conclude that even a brief high-fiber diet intervention may produce profound effects resembling those typically associated with long-term dietary changes beneficial to human health.

Interestingly, a slight but significant decrease of gut community diversity after the short-term diet was observed. This effect of the short-term high-fiber diet appears to be opposite to the correlation between diversity and long-term vegetable consumption clearly seen in our cohort. Other studies linked lower alpha-diversity to immune and metabolic disorders as well as to antibiotic intake [72]. On the other hand, there is evidence to suggest that two weeks of a high-fiber diet may not be enough to affect alpha-diversity [11,13]. A slight drop in alpha-diversity obtained in the current study may reflect the “shock effects” of a relatively rapid change in the spectrum of incoming nutrients, which may transiently disrupt the ecology of the gut community. Another observed facet of microbiota “stress” linked to the transitory period is the slightly but significantly increased abundance of *Staphylococcus* and *Enterobacteriaceae*. Apparently, while the beneficial microbes associated with high-fiber already started to win their niches and extend their presence in two weeks, the disturbance of the ecological network led to the rise of pathobiont and auxotrophic taxa [30,73].

The extent to which gut microbiota reacts to diet interventions was shown to vary across individuals, thus confirming previous observations [74,75,76]. In our study, the degree of response was dependent on initial microbiota composition, but neither on any personalized recommendations nor on any long-term factor revealed by the questionnaire. In the “responders”, a higher abundance of *Bacteroidales* and lower abundance of *Coriobacteriales* and *Clostridiales* was noted at the baseline. Interestingly, bacteria that increased after dieting generally corresponded to the set of taxa underrepresented at the baseline, with a subsequent decrease of the *Bacteroidetes*:*Firmicutes* ratio. Our finding closely resembles observations reported for a Danish cohort of obese and non-obese subjects exposed to a weight-loss diet [75,76]: the microbiota of “responders” was dominated by *Bacteroides*, while “non-responders” had increased proportions of *Blautia*, *Alistipes,* and *Akkermansia*.

**Figure 5:**
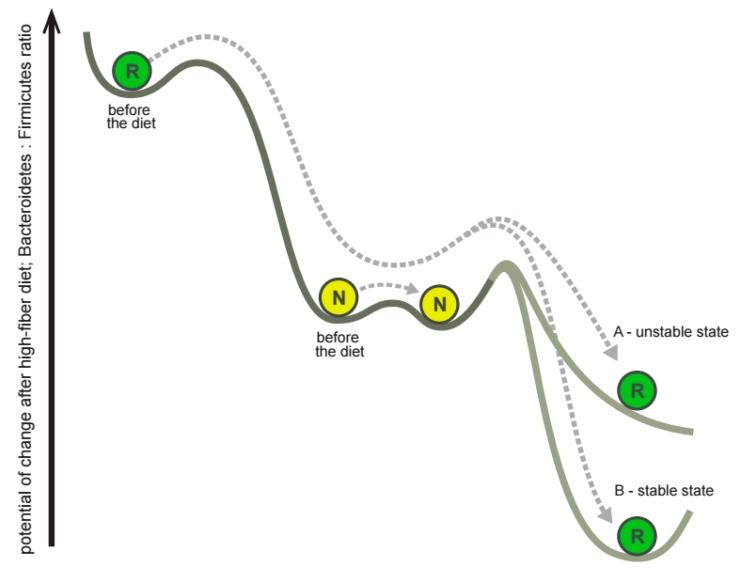
Gut microbiota momentum after the impact of the short-term diet. In the diagram describing the suggested effect, circles denote the location of community structures for typical responders (R) and non-responders (N) before and after the diet in the schematic landscape of possible microbiota configurations. Arrows represent the change of microbiota under the impact of diet.

In “responders”, the microbes overrepresented at the baseline, including *Bacteroidaceae*, became underrepresented after the diet (Figures S12 and S13), while underrepresented microbes, including *Coriobacteriaceae*, became overrepresented (Figures S12 and S13). This “responders”-specific correctional “overshoot” led to a pronounced lowering of the *Bacteroidetes*:*Firmicutes* ratio (Figure 3B,C) after the diet. This observation may reflect a momentum-like property of gut community structure dynamics in the landscape of possible configurations during a high-fiber diet intervention (Figure 5). The *Bacteroidetes*-rich microbiota of “responders” appears to reside in a less stable state than the *Firmicutes*-rich microbiota of “non-responders”, thus making the “responder” more amenable to change. On the contrary, the microbiota of “non-responders” changed slightly upon intervention because of its higher stability. When the microbiota composition of “responders” gains momentum, it moves towards the “non-responders” and even further to reach a state of lower *Bacteroidetes*:*Firmicutes* ratio, normally not accessible to “non-responders”. Whether this acquired community structure remained unstable (marked by A in the Figure 5) or stable (marked by B) is still to be determined. It is also intriguing to examine if other nutritional changes or other types of interventions would result in alternate dynamics and hence alternate stability landscapes.

Although in the present study the dietary recommendations were personalized, ultimately at their core was an advice to consume more high-fiber products. Our observations suggest that a high-fiber diet is expected to produce more pronounced changes in the microbiota of subjects who initially hosted a higher fraction of *Bacteroides*. While this fact could be used to stratify populations before assigning such an intervention, the current results do not allow us to infer directly neither the changes in various microbiota types that will occur after the consumption of specific food products nor their implications for human health. However, our study is one of the first steps towards developing a precision microbiota-tailored personalized diet. It emphasizes that in microbiota surveys of dietary interventions it is important to analyze the interindividual response variability—particularly, to facilitate future meta-analysis. We anticipate further studies on large-scale cohorts from diverse geographic locations who consume specific dietary interventions (preferably, based on the introduction of a single product per study) that will identify responders to these pointwise interventions and further utilize these as a basis to design individual dietary plans. Another important question is related to the concept of response itself. In our study, we assessed it as an overall extent of change in the gut community structure. However, in future studies it can be improved by focusing on the increase of species associated with health, alpha-diversity and/or microbiota resilience—as well as by combining with the physiological parameters of a subject.

Overall, this study expands the current understanding of the extent of the changes in microbiota composition caused by short-term dieting. Advancing a microbiota-targeted diet as a novel modality to be developed in the frame of personalized medicine requires the emergence of early adopters eager to participate in a new trend at the crossroads of translational medicine and citizen science. In this cohort, even a brief, uncontrolled high-fiber diet intervention produced considerable beneficial changes in microbiota. Nevertheless, the observed “shock” effects, although slight, suggest that the duration of microbiota-targeted interventions should be longer than two weeks.

## Supplementary Materials

The following are available online at http://www.mdpi.com/2072-6643/10/5/576/s1.
