# Supplementary material for: Microbiome Responses to an Uncontrolled Short-Term Diet Intervention in the Frame of the Citizen Science Project
Source: Nutrients. 2018 May 8;10(5):576. doi: 10.3390/nu10050576 (PMC5986456; doi:10.3390/nu10050576)
Supplement: Supplementary file 1 [file nutrients-10-00576-s001.zip › Supplementary_information.pdf]

Microbiome Responses to an Uncontrolled Short-Term Diet Intervention in the Frame of the Citizen Science Project

1. Supplementary Methods

1.1. Exclusion criteria

Exclusion criteria were:

- participants under 18 years old
- lack of signed informed consent
- inability to follow the instructions of the study
- positive pregnancy test or lactation
- cancer
- surgeries less than 3 months before the study
- substance abuse

1.2 Lifestyle and food frequency questionnaire

The questionnaire included 96 questions. After grouping of the data, 19 factors were formed:

- Anthropometric factors (n=4): body mass index (BMI), gender, age, presence of chronic diseases;
- Diet (n=9): frequency of consumption of food produced from grains, fruit, vegetables, and meat, dairy products, sweets, alcohol, products rich in starch and vegetable fats;
- Lifestyle (n=4): sleeping hours, number of meals per day, smoking (yes/no);
- Medical factors (n=3): antibiotic treatment within the last 3 months, current intake of any medical drugs, consumption of vitamin dietary supplements within the last month prior to the study (yes/no).

Additionally, two aggregated factors "vegetables and fruit" and "fruit, vegetables and grains" were analyzed.

1.3 Sample preparation: DNA extraction and sequencing

In a sterile box, glass beads (BioSpec Products, USA) with diameter 0.1 mm (300 mg) and 0.5 mm (100 mg) were added to the stool sample (150 mg). Then 1200 µl of a warm lysis buffer (500 mM NaCl, 50 mM Tris-HCl, pH 8.0, 50 mM EDTA, 4% SDS) were added. The mixture was vortexed and homogenized with MiniLys (Bertin Technologies, USA) for 5 minutes. The lysate was incubated at 70°C for 15 minutes. After that the samples were centrifuged for 20 minutes at 1400 rpm. Supernatant was transferred to new tubes and put on ice. The pellet was added to a lysis buffer and the homogenization process was repeated. The supernatants were combined with the addition of 1/10 volume of 20% PVP, vortexed and incubated for 1 minute at the room temperature. The mix was centrifuged for 20 minutes at 14,000 rpm, the supernatant was transferred into new tubes. Two volumes of 96% ethanol and 1/10 volume of 3 M AcNa were added to the supernatant. The mixture was incubated at -20°C for not less than 1 hour, then centrifuged at 14,000 rpm for 20 minutes. The pellet was washed with 80%

ethanol twice, dried in the air and resuspended in 0.1 part of TE-buffer. The RNAse A (5 mg/ml) was added in a ratio of 1 µl of RNAase to 200 µl of TE-buffer; the sample was incubated for 1 hour at 37°C. The obtained DNA solution was stored at -20°C.
The MP Biomedicals 116560200 MP BIOMEDICALS FastDNA™ SPIN Kit for Soil was used to extract DNA from the samples.

The samples of extracted DNA were diluted 500 times. The amplification of a variable region V4 of 16S rRNA gene was performed in 1 round using the primers that in different combinations provide the ability to multiplex 576 samples. Verity (Applied Biosystems) thermocycler was used for amplification under the following PCR cycling conditions:

98°C 30 seconds
30 cycles: 98°C 15 seconds
58°C 15 seconds
72°C 15 seconds
72°C 1 minute
Cooling 4°C

Purification of the PCR products was performed using the Cleanup Mini kit for the extraction of DNA for reagent mixtures (Eurogene).

The concentration of obtained 16S rRNA libraries in the solution was measured with Qubit® fluorometer (Invitrogen, USA) using Quant-iT™ dsDNA High-Sensitivity Assay Kit. The purified amplicons were mixed equimolarly in accordance with the assessed concentrations. The quality of the library prepared for sequencing was assessed using the electrophoresis in agarose gel.

Further sample preparation and sequencing of the pooled sample was performed using MiSeq Reagent Kit v2 (500 cycles) and MiSeq sequencer (Illumina, USA) following the manufacturer's instructions. The primary analysis (demultiplexing) was performed as described by Fadrosh et al. After the quality trimming, the concatenation of the paired reads was performed using SeqPrep software. The final read length was 252 bp.

1.4 Statistical analysis

1.4.1. Primary metagenomic analysis

The obtained reads were analyzed in QIIME v.1.7.0 [1]. The taxonomic analysis was performed by reference-based classification using uclust_ref algorithm and the HITdb v. 1.0 database [2] at the level of operational taxonomic units (OTU) with 97% sequence identity threshold. The classified reads for each sample were randomly rarefied to the same number (5000 reads per sample); the samples with lower coverage were not included in the analysis. Estimation of alpha-diversity for each sample was performed using HITdb database and two indices - chao1 and Shannon. Beta-diversity (pairwise dissimilarity between the gut community structures) was estimated using Bray-Curtis index and generalized UniFrac metric [3]. Read counts of microbial species, genera and families were calculated as the sum of reads classified as OTUs belonging to the respective taxon. Relative abundance of microbial species, genera and families was calculated as the sum of reads classified as any of the OTUs belonging to the taxon divided by the total read count for the sample and

normalized to 100%.

1.4.2. Data preprocessing

Questionnaire data on lifestyle and diet included quantitative (product consumption frequency, etc.), nominal (gender) or logical (antibiotic treatment, etc.) factors. Quantitative factors were tested for having normal distribution (Shapiro-Wilk test, shapiro.test function in R). Factors with non-normal distribution were log-transformed. No strongly correlated (absolute value of Spearman correlation coefficient $|r| > 0.5$) factors were identified, thus all factors were included in the subsequent analyses (full data on correlations are provided in Table S19).

Several samples manifested abnormal community structures, containing high levels of *Enterobacteriaceae*, *Staphylococcaceae* or *Enterococcaceae* as well as one sample having *Bifidobacteriaceae* levels typical of an infant. These samples, together with their paired samples (38 of 430 samples), were excluded from the analysis.

1.4.3. Permatyping - bootstrapping-based cluster analysis of community structures

Permatyping is a bootstrapping-based variation of the enterotyping method for clustering metagenomic samples by their taxonomic composition. Briefly, initially the original enterotyping is performed for the complete set of samples to obtain the clusters. Then, using bootstrapping the samples are classified as stable or unstable - basing on how frequently the sample is placed together with the medoids of the original clusters during random subsampling of the population. Only stable samples are included in the produced permatypes (unstable samples are discarded).

Specifically at the bootstrapping stage, enterotyping is performed 1000 times: each time only a random 50% of the samples from each original enterotype are considered. Then a pairwise dissimilarity matrix for all samples is calculated:

$$D(s1, s2) = \frac{C(s1,s2)}{K(s1,s2)}, \quad (1)$$

where

$s1, s2$ is a pair of samples;

$C(s1, s2)$ - number of iterations resulting in placement of s1 and s2 in the same enterotype;

$K(s1, s2)$ - total number of iterations when s1 and s2 were included in enterotyping.

Each sample s_i is assigned a stability index - the distance $D(s_i, m_k)$ to the medoid of the original cluster of the sample (m_k). The greater the index, the more certain the membership of the sample in the cluster as identified during bootstrapping. As a threshold value, we selected the stability index value corresponding to ASW = 0.1 (stability index = 0.56) in order to keep balance between the robustness of clusters and number of samples included in the analysis (for comparison: ASW for the initial enterotypes was 0.055 – see Figure S20). Samples with stability index above the threshold value are considered stable and assigned a permatype. Totally 250 of 416 samples were stable and were assigned permatypes (Figure S16).

1.4.4. Estimate of variability in gut community structure contributed by experimental factors

In order to compare the effect of various sample processing factors on microbiota composition, we sequenced a number of additional samples. Stool samples were collected from 7 additional volunteers who did not change their diet - before and after 2-week period (Table S21). Two of these subjects additionally collected stool samples each 3 days (5 samples per subject totally). For 2 samples from the same subject, DNA extraction was independently performed twice (followed by sequencing). Five samples from different subjects were collected into 2 tubes each. For samples of 31 subjects, sequencing libraries were prepared twice (followed by sequencing). Pairwise dissimilarity between the taxonomic composition profiles obtained from these metagenomes was used to sort the contribution of various factors in comparison with the effect of 2-week diet.

2. Supplementary Figures

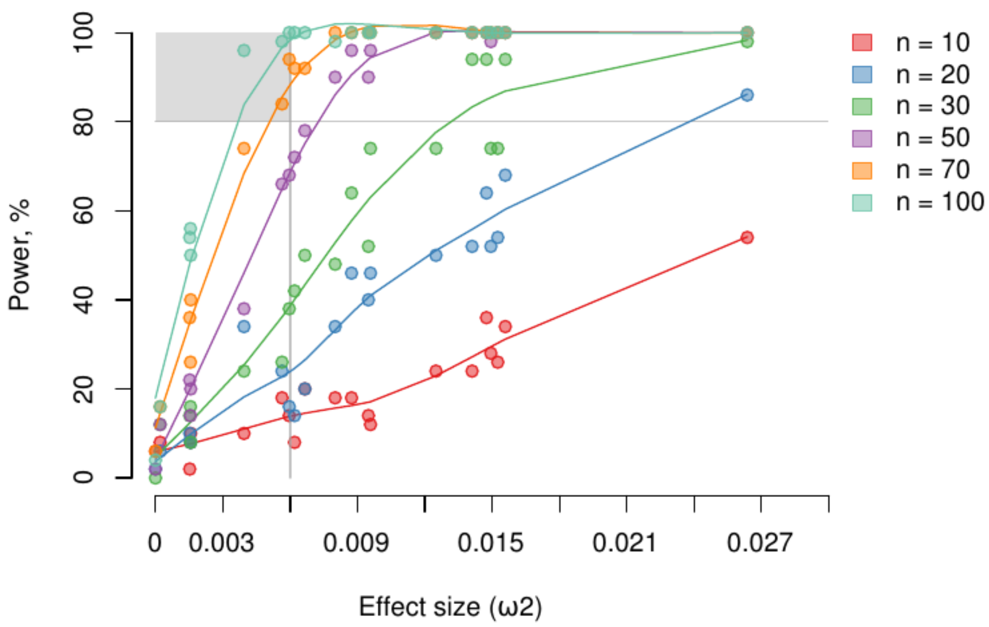

Figure S1 - Power analysis for PERMANOVA short-term diet impact test. Grey area indicates sample sizes (n) sufficient to detect the effect size of $\omega^2 \leqslant 0.006$ with statistical power of $\geq 80\%$. The analysis was performed using micropower R package.

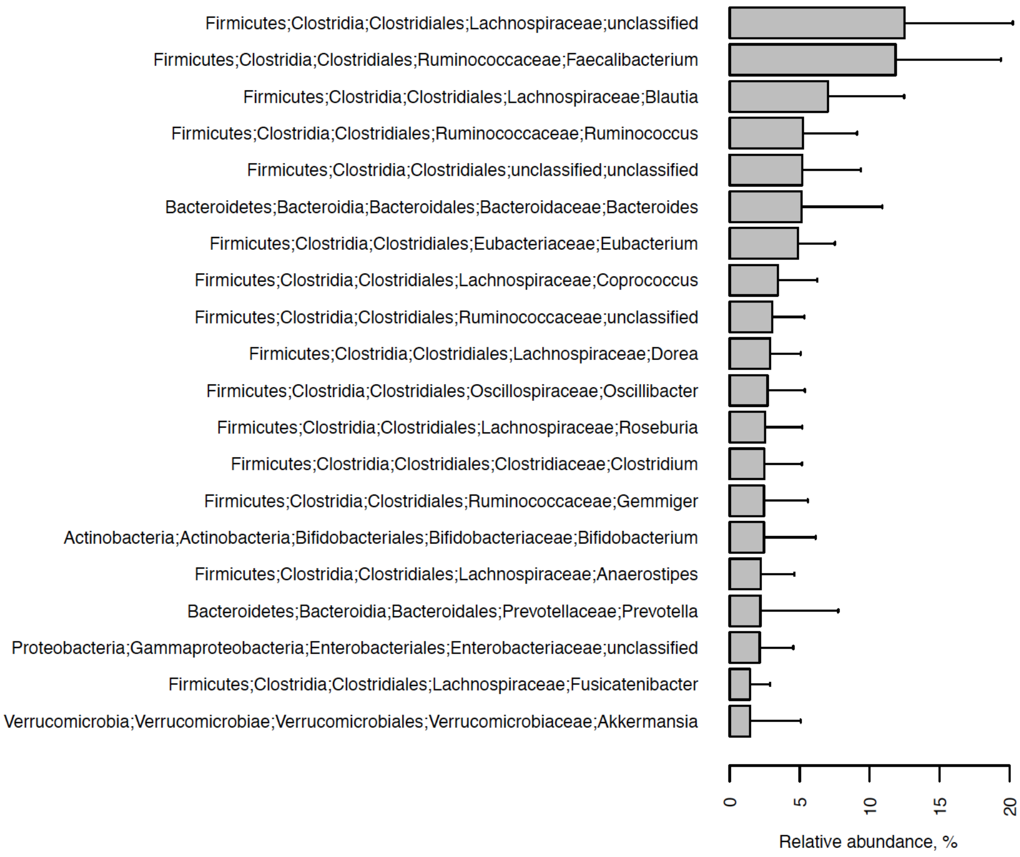

Figure S2 - Major microbial genera for the complete set of metagenomes. Relative abundance for 20 most abundant genera is shown (mean±s.d., n=430 samples).

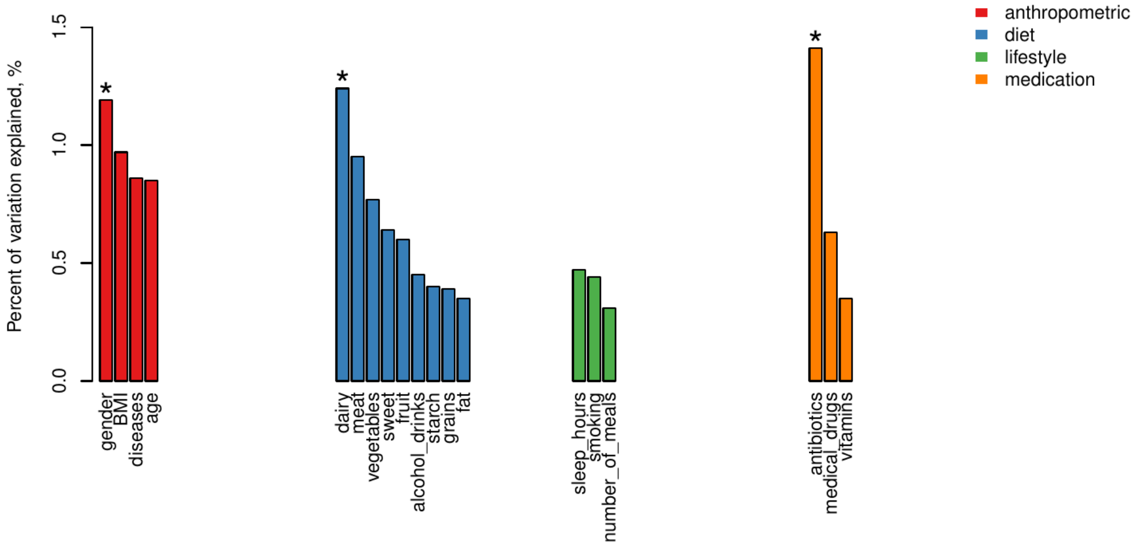

Figure S3 - Percent of variation in taxonomic composition (calculated via Generalized Unifrac dissimilarity measure) explained by various groups of factors from the questionnaire. Asterisks denote significant associations (FDR-adjusted $p < 0.05$).

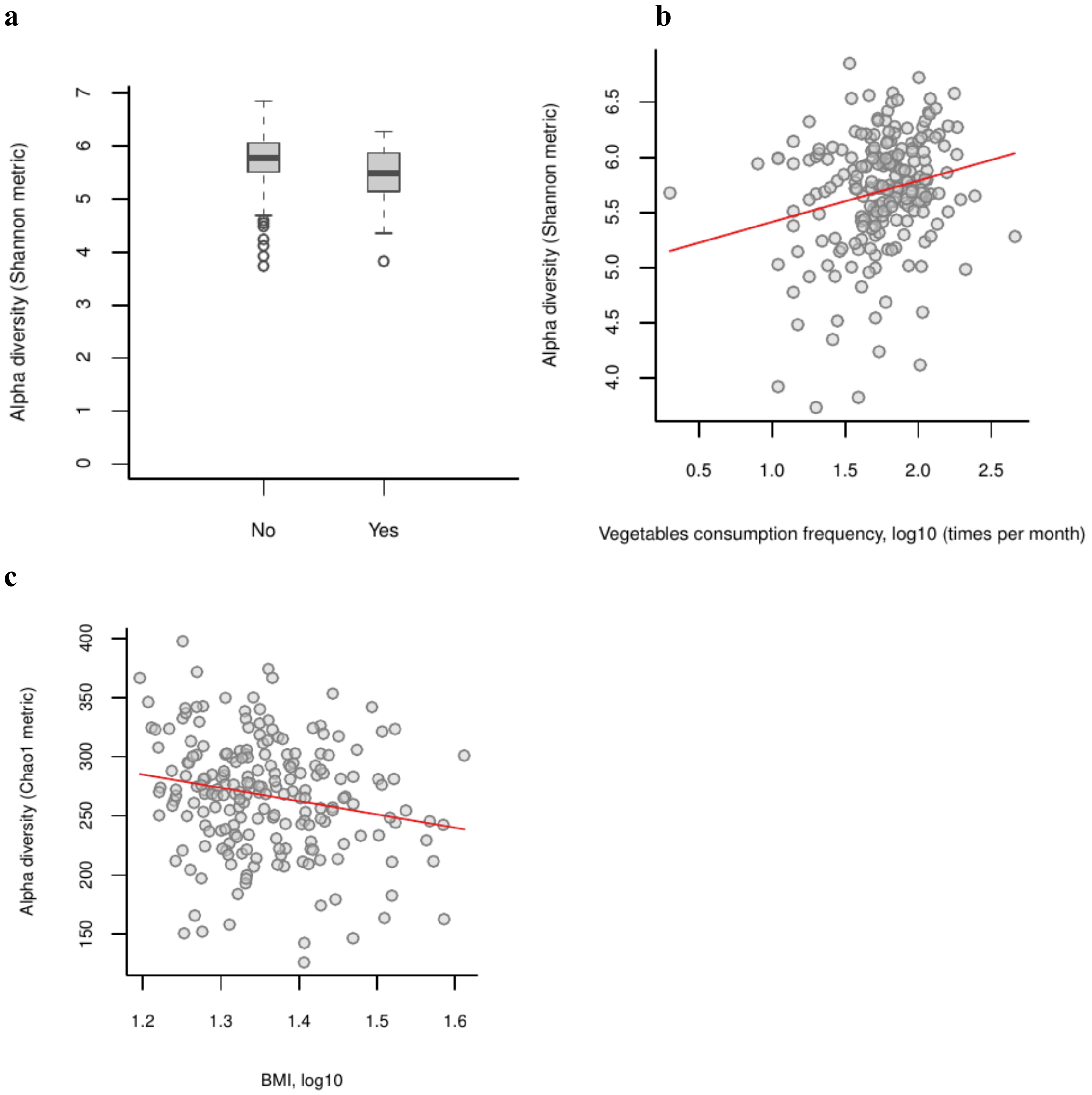

Figure S4 - Factors from the questionnaire significantly associated with baseline microbiota alpha-diversity (Spearman correlation $p < 0.05$).
(**a**) Antibiotic treatment within 3 months before the analysis (n = 207). (**b**) Vegetable consumption frequency (n = 207). (**c**) BMI (n = 207).

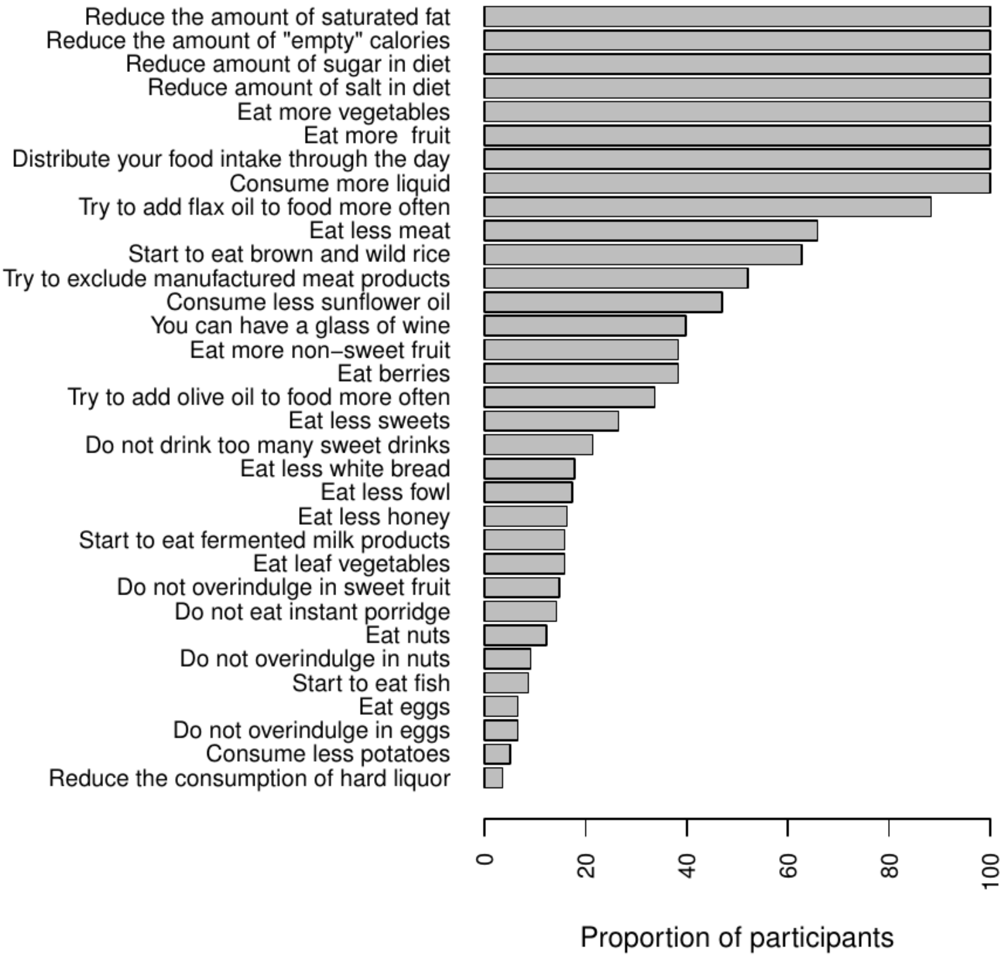

Figure S5 - Frequency of assignment for each personalized recommendation across the cohort (n=215 subjects).

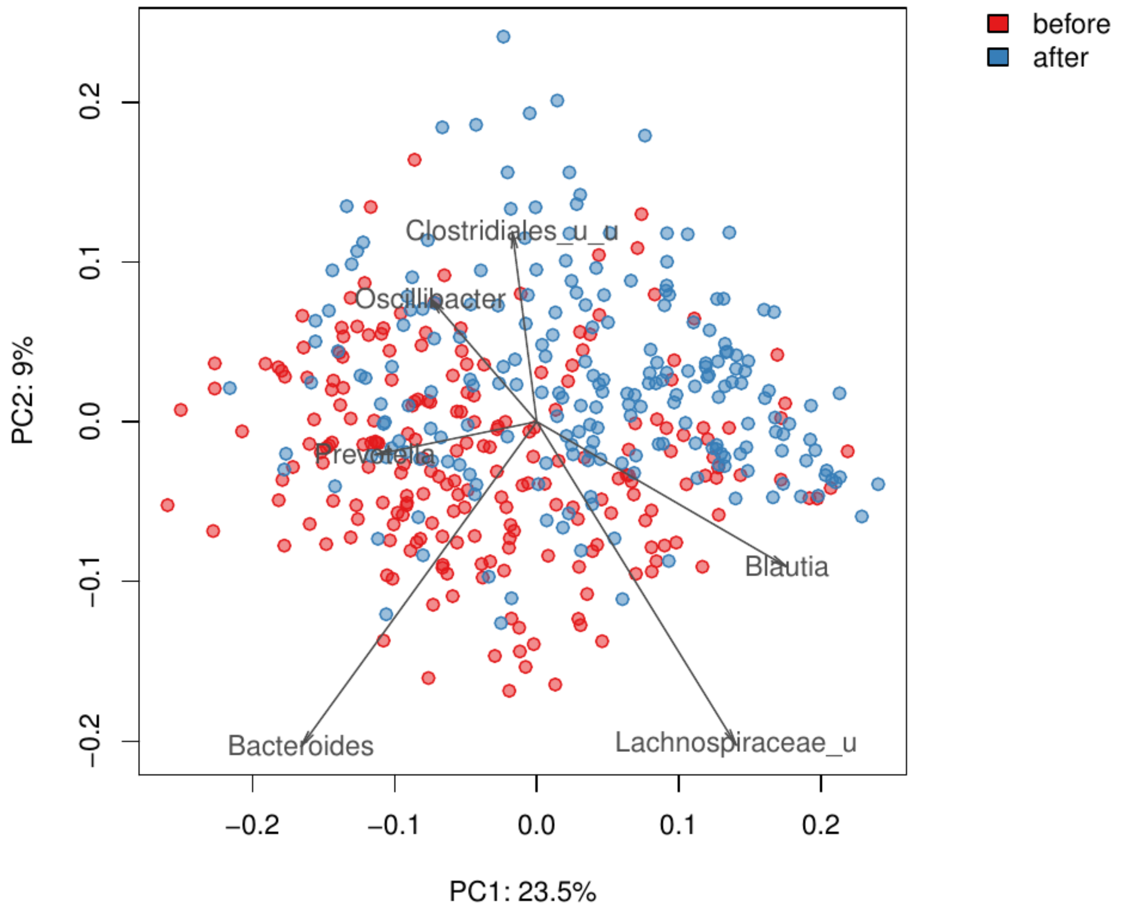

Figure S6 - General shift in microbiota taxonomic composition after the diet intervention. PCoA plot is constructed using generalized UniFrac distance). The samples before and after the intervention are shown in red and blue, respectively.

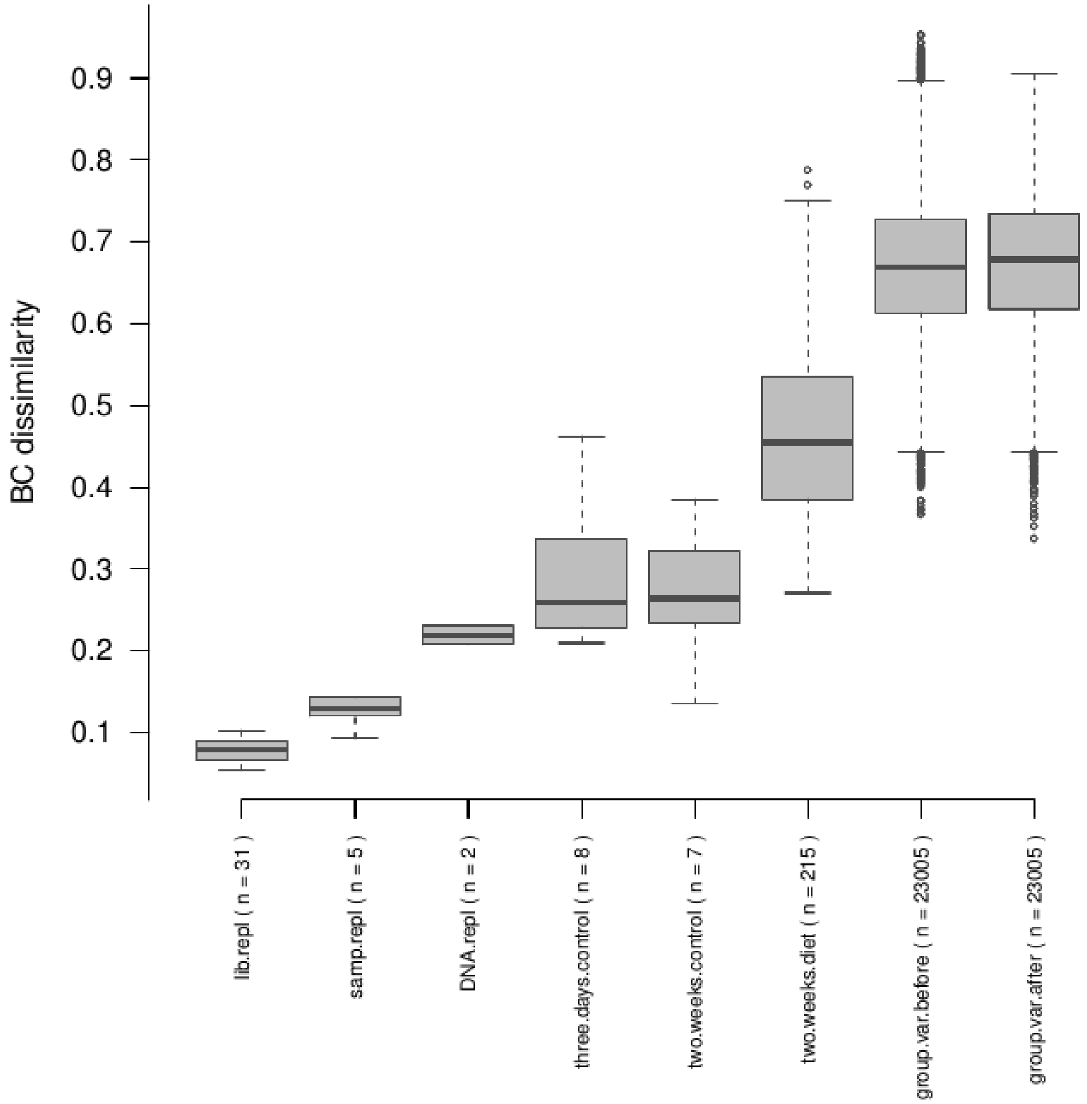

Figure S7 - Contribution of experimental factors to the microbiota composition profiles. Shown are the distributions of pairwise dissimilarity within subgroup (twoness is stated explicitly; Bray-Curtis measure); n is the number of comparisons in each group. Labels:

- lib.repl - technical replicates at the level of library preparation (60 samples from 30 subjects), paired comparison;
- sample.repl - technical replicates at the level of sample collection (10 samples from 5 subjects), paired comparison;
- DNA.repl - technical replicates at the level of DNA extraction (2 samples from 1 subject), paired comparison;
- three.days.control - samples from subjects without change of diet taken 3 days apart (10 samples from 2 subjects), paired comparison;
- two.weeks.control - samples from subjects without change of diet taken 2 weeks apart (14 samples from 7 subjects), paired comparison;
- two.weeks.diet - samples before and after the diet for main cohort (430 samples from 215 subjects), paired comparison (430 samples from 215 subjects);
- group.var.before - groupwise variation for main cohort at the baseline (215 samples from 215 subjects), unpaired comparison;
- group.var.after - groupwise variation for main cohort after the intervention (215 samples from 215 subjects), unpaired comparison.

a

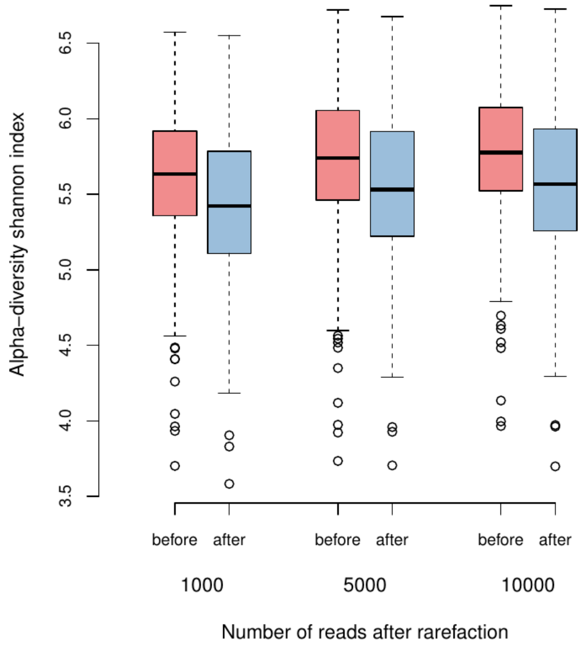

b

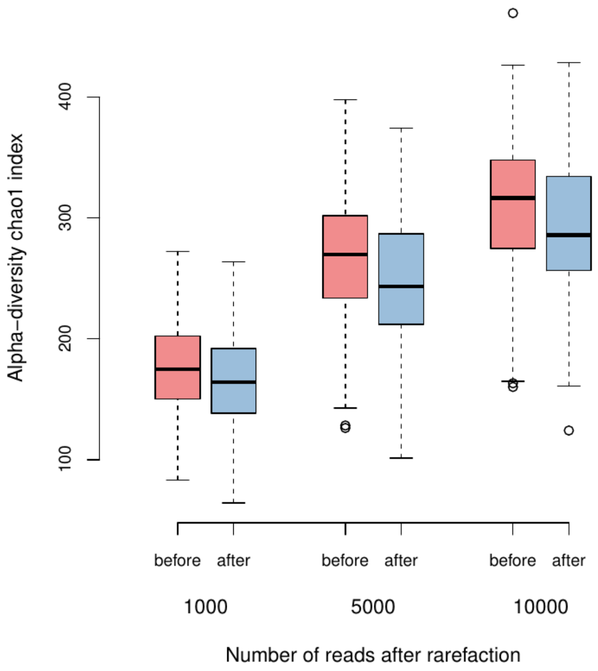

c

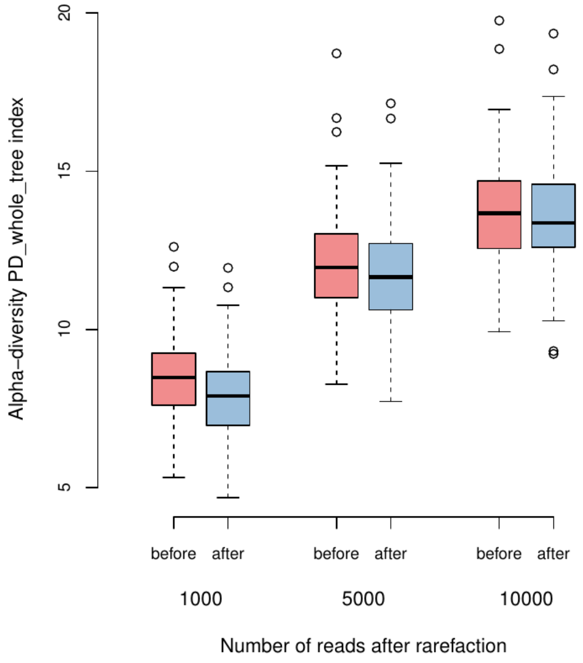

Figure S8 - Comparison of alpha-diversity before and after dietary intervention calculated using several metrics and read rarefaction thresholds. Rarefaction was performed at the levels of 1000, 5000 and 10000 reads per sample (n = 430). **(a)** Shannon

index, **(b)** chao1, **(c)** Phylogenetic diversity (PD_whole_tree).

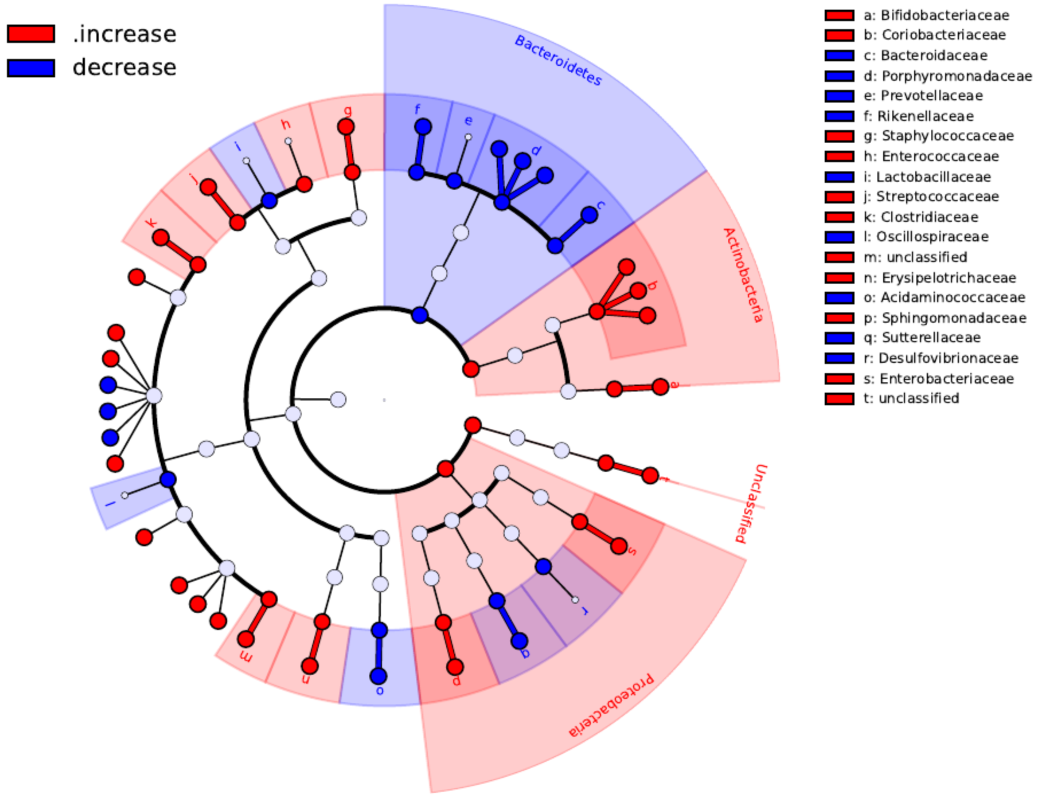

Figure S9 - Validation of major changes in the gut community structure of the volunteers after following the dietary recommendations using ALDEx2 method. Red branches of the cladogram denote the taxa that were increased in abundance, while the blue ones - decreased. Significance criterion: $p<0.05$ in ALDEx2 method and $\log_{10}$ of the effect size>2 in LEfSe method (n=430 paired samples).

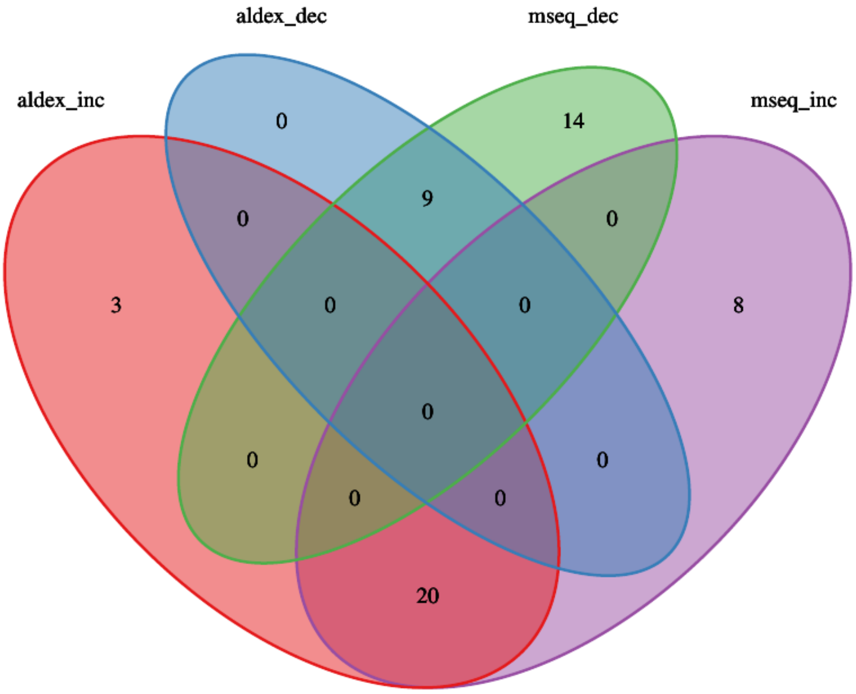

Figure S10 - Vienn Diagram for comparison between metagenomeSeq (mseq) and ALDEx2 (aldex) results for differential abundance analysis of short-term diet effect on genus level. "_dec" means decreased taxa, and "_inc" meas increased

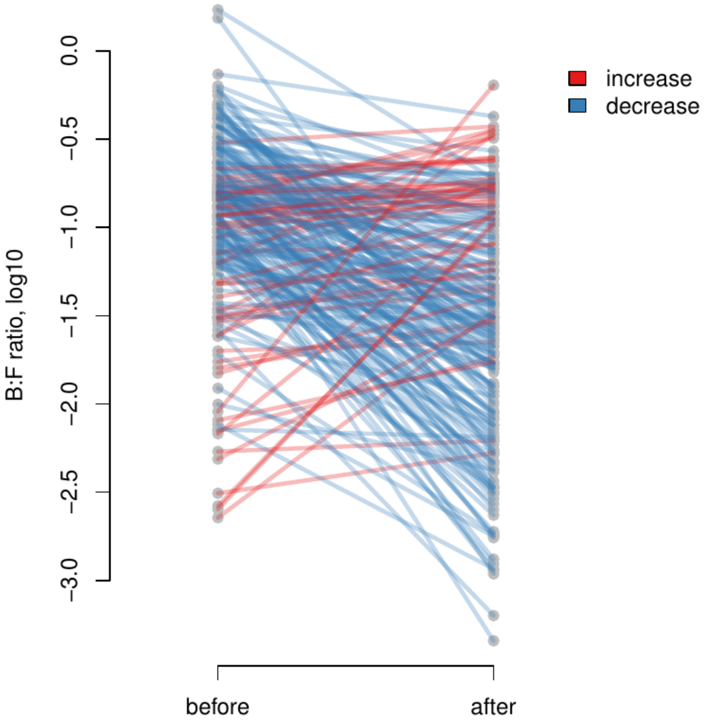

Figure S11 - Comparison of Bacteroidetes : Firmicutes ratio before and after following the dietary recommendations. Each point on the left and right sides of the slopegraph correspond to the samples collected before and after the diet, respectively. Paired samples from the same subject are connected with a line the color of which shows the direction of change for the ratio.

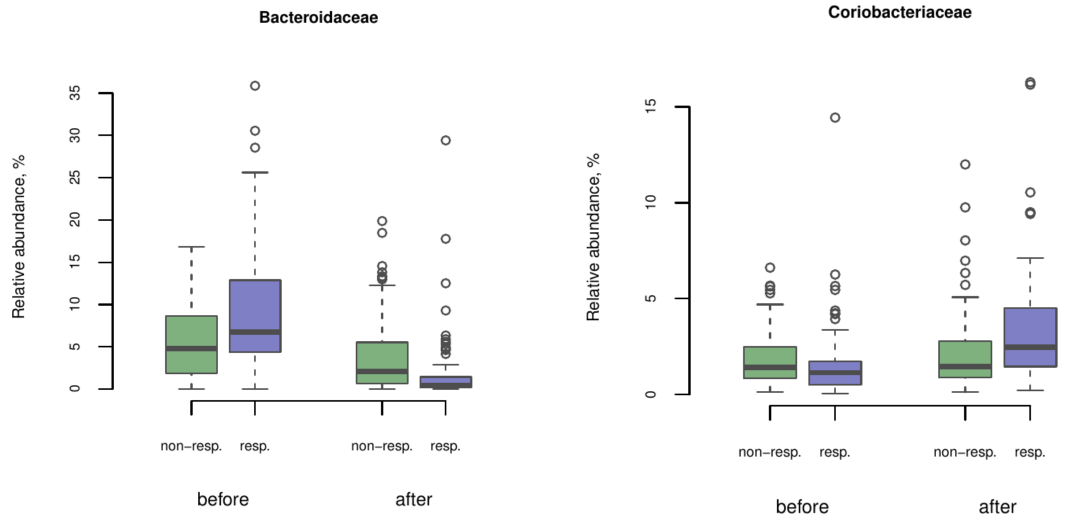

Figure S12 - Examples of major increased and decreased microbial families in microbiota of responders (resp. n=85) and non-responders (non-resp. n=130) after the dietary intervention. (**a**) Increased: Coriobacteriaceae. (**b**) Decreased: Bacteroidaceae.

a

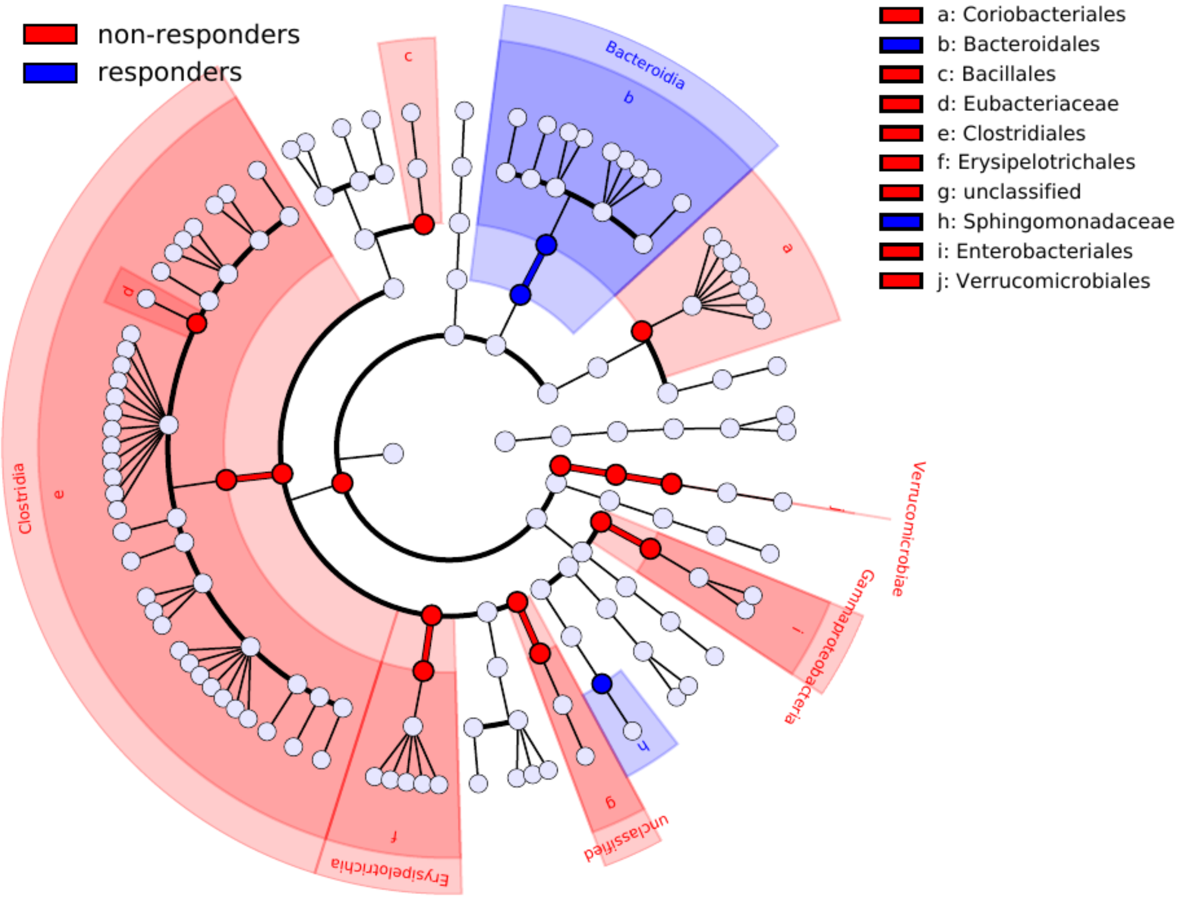

b

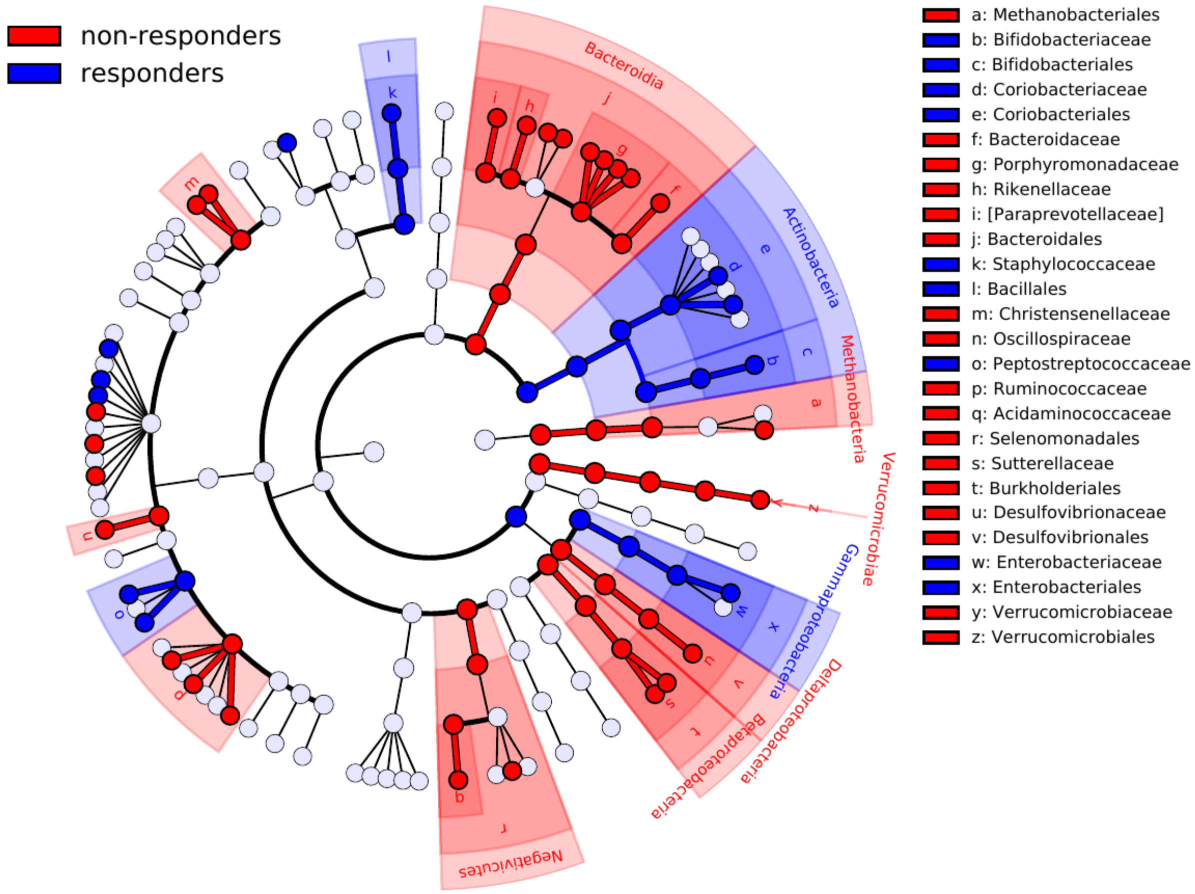

Figure S13 - Microbial taxa differentially abundant in gut microbiota of responders and non-responders. (a) Before the diet. **(b)** After the diet. The plots were constructed using LEfSe. Significance criterion: $p<0.05$ in metagenomeSeq model and $\log_{10}$ of the effect size>2 in LEfSe method (n=215 pairs of samples).

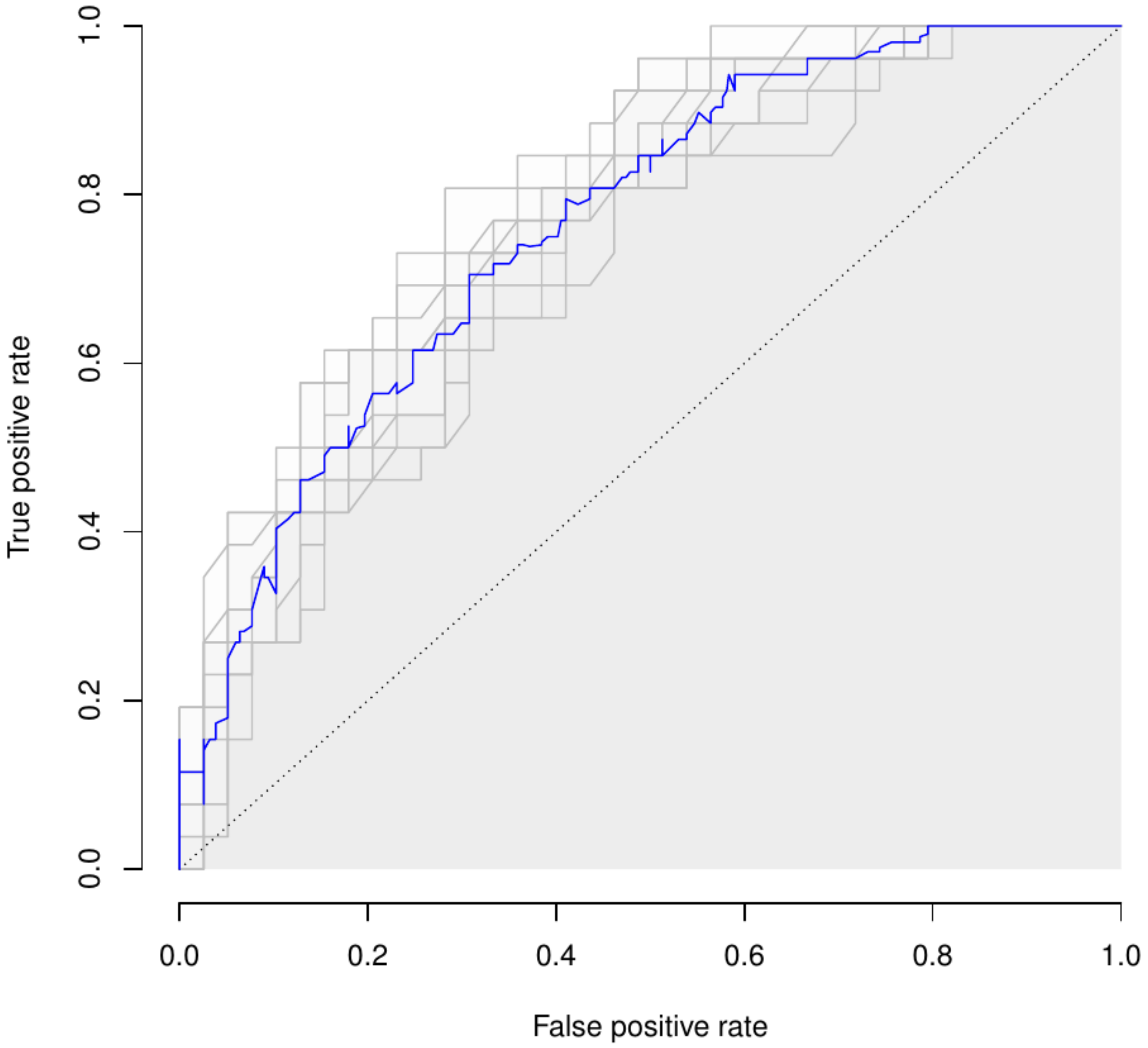

Figure S14 - Receiver operating characteristic (ROC) curves for responders/non-responder classifier group based on baseline microbiota composition at the species level. Grey lines correspond to performance in 10 random cross-validation iterations (train N=150, test N=65). Blue line shows the mean ROC curve. Dotted black line corresponds to a random predictor.

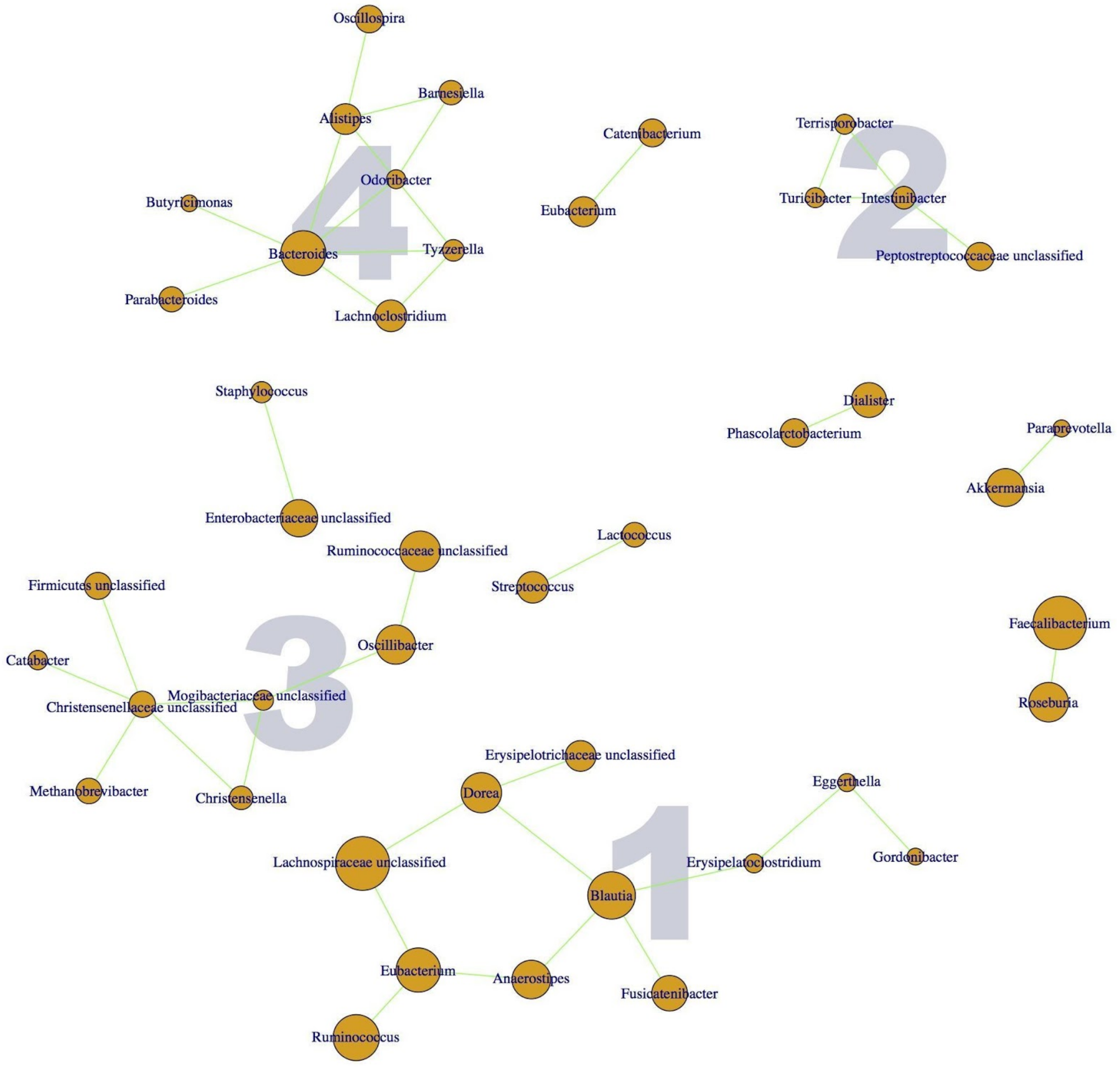

Figure S15 - Co-occurrence graph of microbial genera. The numbered connected components correspond with 4 cooperatives. Size of vertices is proportional to the average relative abundance of the genera in all metagenomes.

1 - Lachnospiraceae-dominant cooperative.

2 - Peptostreptococcaceae-dominant cooperative.

3 - Ruminococcaceae-dominant cooperative.

4 - Bacteroides-dominant cooperative.

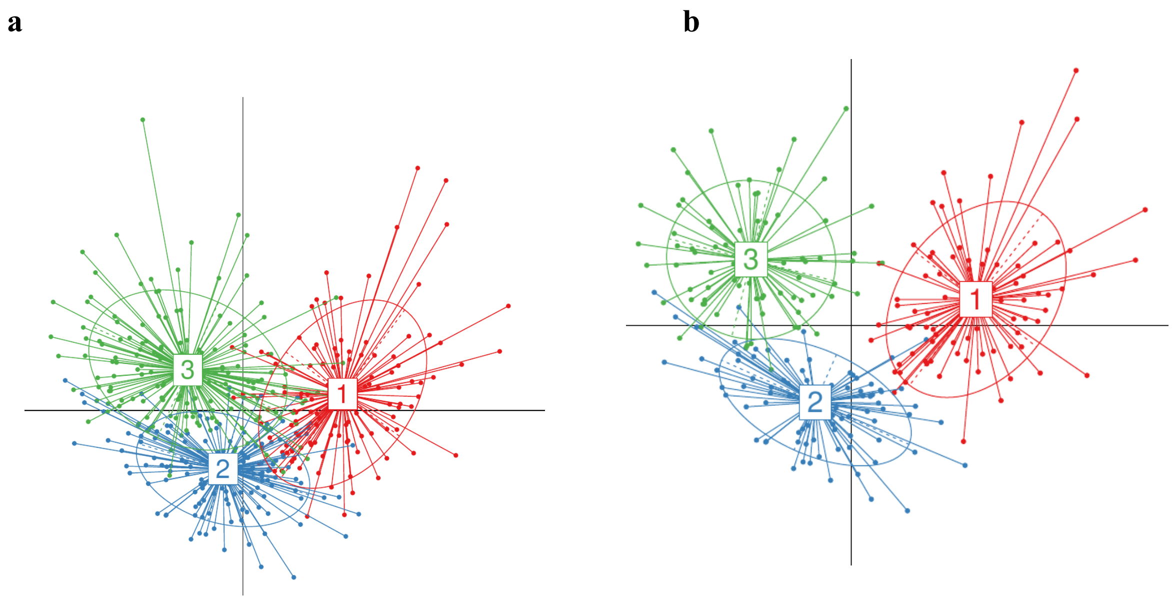

Figure S16 - Comparison of enterotyping and permatyping results using between-class analysis (BCA) plots. (**a**) Enterotypes. (**b**) Permatypes (unstable samples are not shown, according to the definition of permatypes).

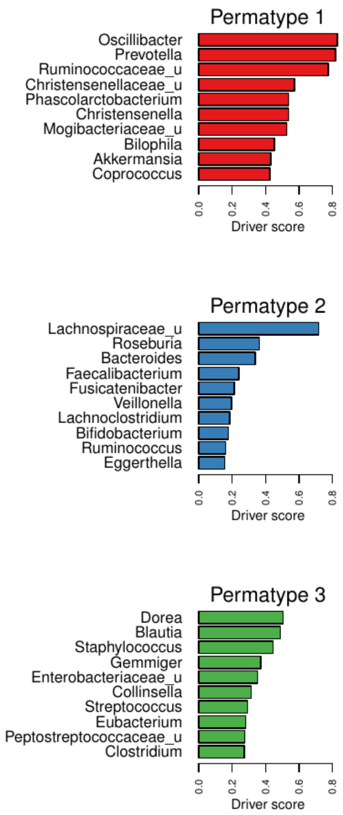

Figure S17 - Distinctive microbial genera for the 3 permatypes. The set of drivers and their scores were calculated using between-class analysis (BCA) as in the original enterotyping procedure.

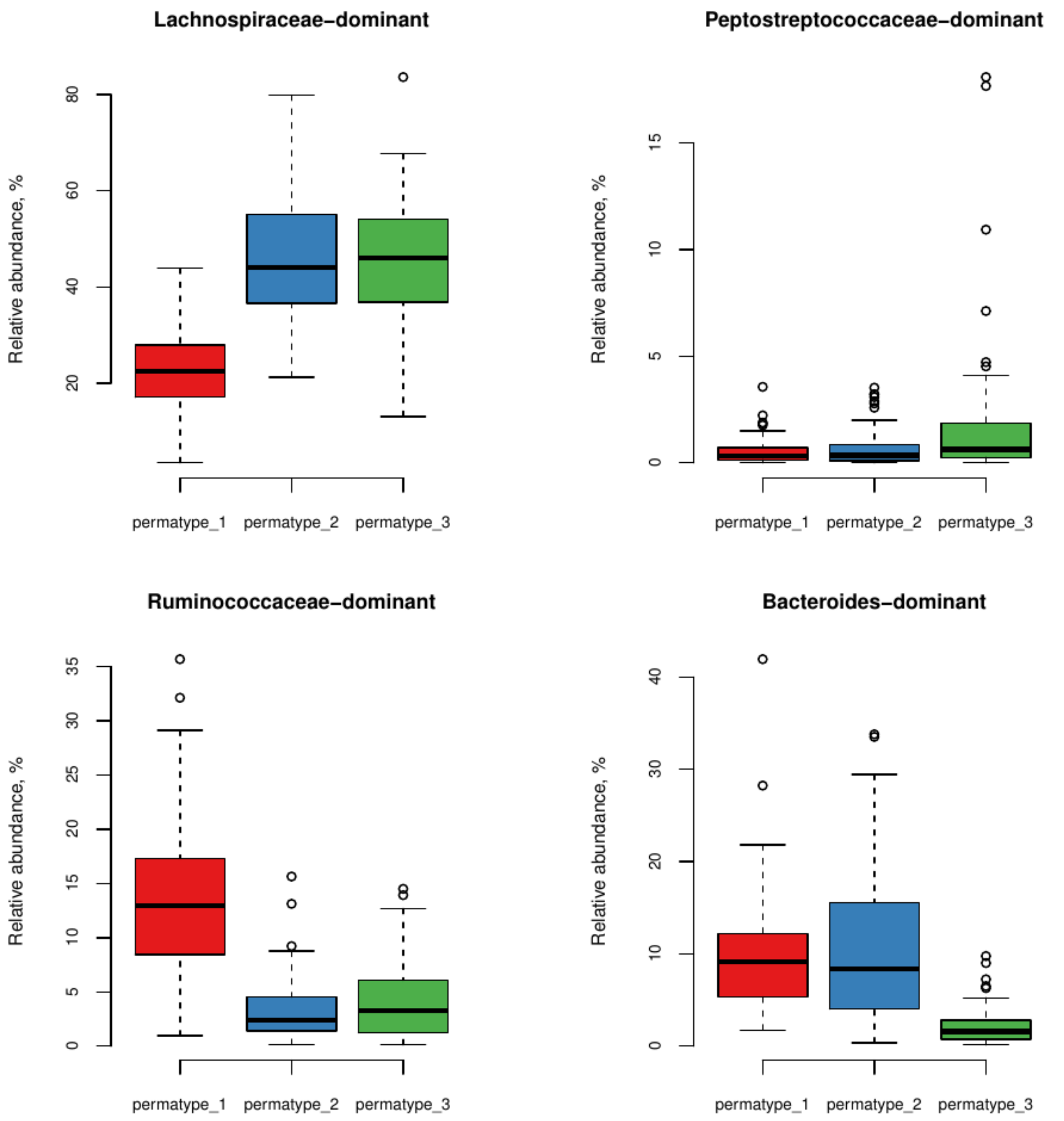

Figure S18 - Distribution of relative abundance of microbial cooperatives across permatypes (first permatype n=83, second permatype n=86 and third permatype n=81).

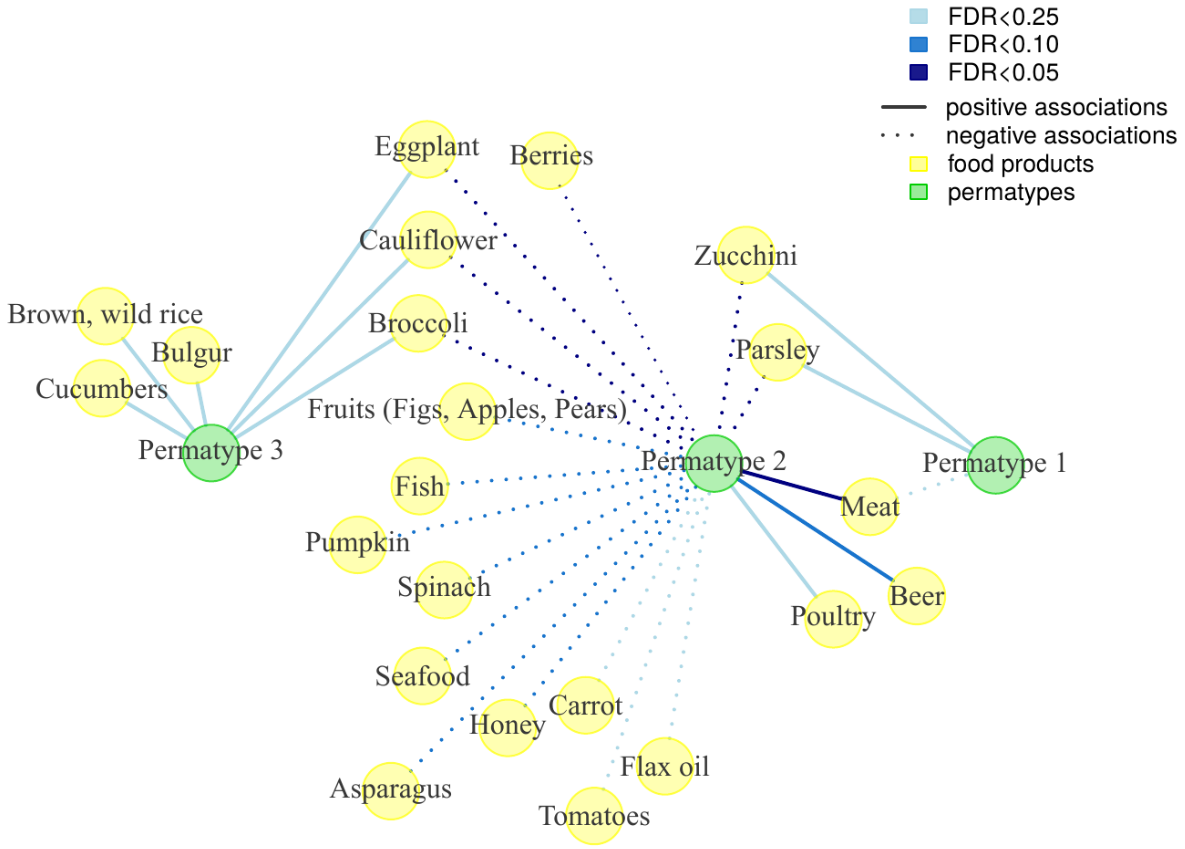

Figure S19 - Associations between permatypes and food products consumption frequency. Dark blue lines denote significant associations (FDR adjusted p < 0.05) and light blue lines - trends (FDR adjusted p < 0.25).

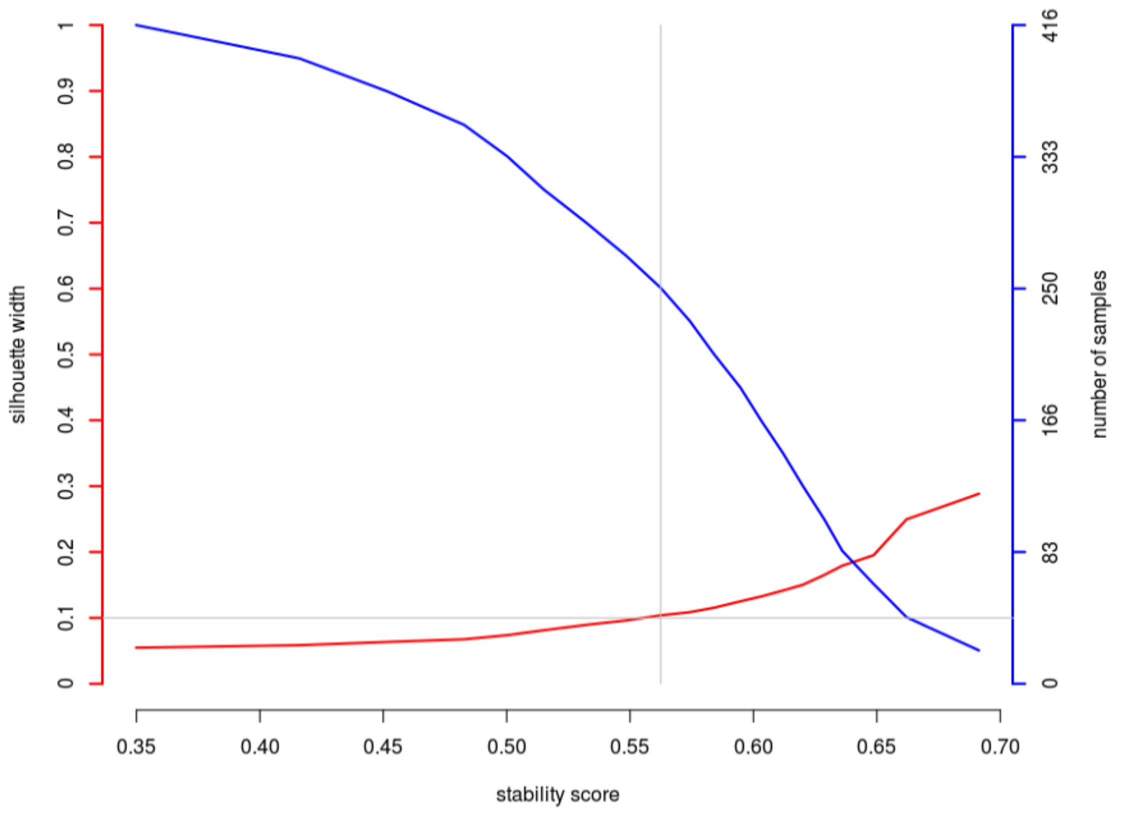

Figure S20 - Relation of average silhouette width and number of samples included in permatypes. Grey lines show the selected stability threshold that corresponds to ASW = 0.1.

3. Supplementary Tables

Table S1 - Dietary and lifestyle questionnaire results (including the original data and data for aggregated product groups).

Table S2 - General and personolized dietary recommendations wich were given to participants.

Table S3 - Connection between questionnaire results and dietary recommendations.

Table S4 - Relative OTU abundance for all samples.

Table S5 - Statistics for questionnaire results.

Table S6 - Associations between long-term diet and microbiota composition (results of MaAsLin).

Table S7 - Identifiers of gut metagenomes paired by subject. For participants who collected a sample only before the diet intervention, “not_defined” value is given for the second time point (after the intervention).

Table S8 - Changes in relative abundance of microbial taxa after dietary intervention. The analysis was performed using metagenomeSeq. For each taxon, the columns “Beta” and “Eff. size” contain the values of linear model coefficient and effect size calculated using LEfSe.

Table S9 - Changes in relative abundance of microbial taxa after dietary intervention. The analysis was performed using ALDEx2. For each taxon, the columns “Beta” and “Eff. size” contain the values of linear model coefficient and effect size calculated using LEfSe.

Table S10 - KEGG pathways for which their relative abundance changed significantly after dietary intervention. The values in the “p_adj_up" column denote the significance for the pathways increased in abundance after the diet, and "p adj dist.dir.dn" - for the decreased ones. The columns “Genes total”, “Genes up”, “Genes down” show the total number of genes in the pathway as well as the numbers of the genes increased and decreased after the diet, respectively.

Table S11 - KEGG modules for which their relative abundance changed significantly after the diet intervention. The columns are similar to the ones present in the table for KEGG pathways.

Table S12 - Distribution of subjects between the clusters of responders and non-responders. Paired sample identifiers for each subject are shown.

Table S13 - Microbial taxa differentially abundant in gut microbiota of responders and non-responders before the diet (calculated using metagenomeSeq).

Table S14 - Microbial taxa differentially abundant in gut microbiota of responders and non-responders after the diet (calculated using metagenomeSeq).

Table S15 - Mean performance characteristics of random forest classifier for prediction responder/non-responder status at different taxonomic levels.

Table S16 - Composition of the microbial cooperatives.

Table S17 - Distribution of samples between the permatypes.

Table S18 - Associations between permatypes and food consumption frequency. The values in the “P-value (greater)” column correspond to the alternative hypothesis that consumption of the product is greater for the members of this permatype than for the other subjects, while the “P-value (lower)” column is for the alternative hypothesis that it is lower.

Table S19 - Pairwise Spearman correlation between factors from the questionnaire.

Table S20 - Associations between each of the factors included in the metadata and general microbiota composition. Subjects who reported administration of medical drugs were excluded from the analysis.

Table S21 - Description of the additional samples analyzed to estimate the variability in

gut community structure profile contributed by different experimental factors.

4. Supplementary Notes

4.1. Comparison of questionnaire data with microbiota composition

The questionnaires were completed by 238 of 260 subjects (91%). As gluten and lactose intolerance might be linked to altered microbiota composition and fractions of subjects who positively answered these questions were low - 3% and 10%, respectively (8 and 23 of 238 subjects) - these samples were excluded from the baseline analysis. In total, multifactor analysis was performed for 207 subjects.

98 of the subjects (47%) reported medical drug use. Lack of detailed information on the drug type (besides antibiotics reported separately) prevented the consideration of this item as substantial because the impact of drugs on gut microbiota varies widely. For this reason, the whole multifactor analysis was repeated without these samples (see Table S20). Likely due to the decreased statistical power after this 2× reduction of sample size, we only detected a smaller number of associations: the factors linked to general composition included gender and fruit consumption frequency (explained variance using Bray-Curtis measure 1.73% and 1.63%, respectively; FDR-adjusted $p < 0.1$); no links were found using generalized UniFrac measure, also no links with alpha-diversity were found.

Supplementary References

1. Caporaso, J.G.; Kuczynski, J.; Stombaugh, J.; Bittinger, K.; Bushman, F.D.; Costello, E.K.; Fierer, N.; Pena, A.G.; Goodrich, J.K.; Gordon, J.I.; et al. QIIME allows analysis of high-throughput community sequencing data. Nature methods 2010, 7, 335. doi:10.1038/nmeth.f.303.
2. Ritari, J.; Salojärvi, J.; Lahti, L.; de Vos, W.M. Improved taxonomic assignment of human intestinal 16S rRNA sequences by a dedicated reference database. BMC genomics 2015, 16, 1056. doi:10.1186/s12864-015-2265-y.
3. Chen, J.; Bittinger, K.; Charlson, E.S.; Hoffmann, C.; Lewis, J.; Wu, G.D.; Collman, R.G.; Bushman, F.D.; Li, H. Associating microbiome composition with environmental covariates using generalized UniFrac distances. Bioinformatics 2012, 28, 2106–2113. doi:10.1093/bioinformatics/bts342.
